# Structural characterization of Class 2 OLD family nucleases supports a two-metal catalysis mechanism for cleavage

**DOI:** 10.1093/nar/gkz703

**Published:** 2019-08-10

**Authors:** Carl J Schiltz, April Lee, Edward A Partlow, Christopher J Hosford, Joshua S Chappie

**Affiliations:** Department of Molecular Medicine, Cornell University, Ithaca, NY 14853, USA

## Abstract

Overcoming lysogenization defect (OLD) proteins constitute a family of uncharacterized nucleases present in bacteria, archaea, and some viruses. These enzymes contain an N-terminal ATPase domain and a C-terminal Toprim domain common amongst replication, recombination, and repair proteins. The *in vivo* activities of OLD proteins remain poorly understood and no definitive structural information exists. Here we identify and define two classes of OLD proteins based on differences in gene neighborhood and amino acid sequence conservation and present the crystal structures of the catalytic C-terminal regions from the *Burkholderia pseudomallei* and *Xanthamonas campestris* p.v. *campestris* Class 2 OLD proteins at 2.24 Å and 1.86 Å resolution respectively. The structures reveal a two-domain architecture containing a Toprim domain with altered architecture and a unique helical domain. Conserved side chains contributed by both domains coordinate two bound magnesium ions in the active site of *B. pseudomallei* OLD in a geometry that supports a two-metal catalysis mechanism for cleavage. The spatial organization of these domains additionally suggests a novel mode of DNA binding that is distinct from other Toprim containing proteins. Together, these findings define the fundamental structural properties of the OLD family catalytic core and the underlying mechanism controlling nuclease activity.

## INTRODUCTION

Phosphoryl transfer reactions are critical for the synthesis and processing of nucleic acids (1). DNA and RNA polymerization, nuclease degradation, RNA splicing, and DNA transposition all proceed via the same general reaction scheme involving (i) an SN2 nucleophilic attack on the scissile phosphodiester bond, (ii) the formation of a pentavalent transition state and (iii) cleavage of the scissile bond leading to stereo inversion of the scissile phosphate and release of the leaving group (2). These steps depend on the presence of a basic moiety to activate the nucleophile, a general acid to protonate the leaving group, and the presence of positively charged groups to stabilize the developing negative charge in the transition state (3,4). The observed catalytic activity of RNA (5,6) coupled with the presence of two metal ions in the refined structures of alkaline phosphatase (7) and the Klenow fragment with DNA (8) led to the generalized mechanistic hypothesis that metals can substitute for protein side chains in phosphoryl transfer reactions and act as the required general acid and base (9). In this scheme, one metal (metal A) deprotonates the nucleophile while the other (metal B) stabilizes the pentavalent transition state intermediate (2). Despite the prevalence of this mechanism, the number of metal cofactors can vary among different enzyme families. Many homing endonucleases, for example, function using one metal (10,11) while *in crystallo* catalytic studies of human DNA polymerase η reveal an essential catalytic role for a third metal during DNA synthesis (12). Structural characterization of phosphoryl-hydrolases is therefore necessary for understanding the underlying catalytic strategy employed in each case.

Topoisomerases, DnaG primases, gyrases, RecR recombination proteins and 5S rRNA maturases share a conserved catalytic domain that mediates metal-dependent nicking and cleavage of nucleic acid substrates (13,14). This **Top**oisomerase/**prim**ase (Toprim) domain consists of a four-stranded parallel β-sheet sandwiched between two pairs of α-helices and contains three key sequence motifs: an invariant glutamate located in the α1-β1 loop, an invariant glycine following β2, and a conserved DxD motif between α3 and β3 (13,15). Crystallography and mutagenesis have shown the conserved E and DxD motif to be critical for metal binding and catalytic activity in multiple contexts (14,16–18). Additional active site components vary between Toprim family members based on specific functional requirements. Toposiomerases and gyrases contain a catalytic tyrosine that forms a covalent linkage with the DNA (15,19) whereas DnaG primases have extra acidic residues that coordinate multiple metals needed for nucleotide binding and polymerase activity (16,17,20). While most Toprim proteins play important roles in DNA replication, recombination, and repair, recent structural studies revealed the CWB2 cell wall-anchoring module of *Clostridium difficile* proteins Cwp8 and Cwp6 also contains a Toprim fold (21). These domains, however, lack the conserved metal binding side chains and form trimers that act in a purely structural capacity.

**O**vercoming **l**ysogenization **d**efect (OLD) proteins constitute a family of uncharacterized enzymes that contain a predicted N-terminal ATPase domain and C-terminal Toprim domain (13,22). Much of our present understanding of OLD function derives from bacteriophage P2 genetic and biochemical studies. The P2 *old* gene product interferes with bacteriophage λ growth in P2 lysogens, kills *Escherichia coli recB* and *recC* mutants following P2 phage infection, and causes increased sensitivity of P2 lysogens to X-ray irradiation (23–25). These effects appear to be accompanied by a partial degradation of tRNA molecules and inhibition of protein synthesis (26,27). P2 OLD purified as a maltose binding protein fusion exhibits 5′-3′ exonuclease cleavage of DNA and ribonuclease activity *in vitro* (28). Recent genetic studies indicate that the *Salmonella typhimurium old* gene becomes critical under certain growth conditions like temperature stress (29), but its mechanism of action and normal physiological functions remain a mystery. Nothing is known about the activities of other homologs and there are currently no structures of OLD proteins.

Here we identify and define two classes of OLD proteins based on differences in gene neighborhood and amino acid sequence conservation. We purify and characterize the Class 2 OLD proteins from *Burkholderia pseudomallei* (Bp) and *Xanthamonas campestris* p.v. *campestris* (Xcc) and present the crystal structures of their catalytic C-terminal regions at 2.24 and 1.86 Å resolution respectively. The structures show a two-domain arrangement containing a Toprim domain with altered architecture and a unique helical domain. Conserved side chains contributed by both domains coordinate two bound magnesium ions in the active site of Bp OLD, which are absolutely required for nuclease activity. The geometry of this catalytic machinery supports a two-metal catalysis mechanism for cleavage and shows unexpected structural conservation with the active sites of DnaG primases and bacterial RNase M5 maturases. The spatial organization of these domains additionally suggests a novel mode of DNA binding that is distinct from other Toprim containing proteins. Together, these findings define the fundamental structural properties of the OLD family catalytic core and the underlying mechanism controlling nuclease activity.

## MATERIALS AND METHODS

### Expression and purification of OLD constructs

Full-length (Xcc^FL^, residues 1–595; Bp^FL^, residues 1–594) and C-terminal region (Xcc^CTR^, residues 374–595; Bp^CTR^, residues 374–594) OLD constructs were cloned into pET21b, introducing a C-terminal 6xHis tag for purification. Constructs were transformed into BL21(DE3) cells, grown at 37°C in Terrific Broth to an OD_600_ of 0.7–0.9, and then induced with 0.3 mM IPTG overnight at 19°C. Cells were harvested, washed with nickel load buffer (20 mM HEPES pH 7.5, 500 mM NaCl, 30 mM imidazole, 5% glycerol (v/v), and 5 mM β-mercaptoethanol), and pelleted a second time. Pellets were typically flash frozen in liquid nitrogen and stored at −80°C.

Pellets from 500 ml cultures were thawed and resuspended in 30 ml of nickel load buffer supplemented with 10 mM PMSF, 5 mg DNase, 5 mM MgCl_2_, and a Roche complete protease inhibitor cocktail tablet. Lysozyme was added to 1 mg/ml and the mixture was incubated for 15 min rocking at 4°C. Cells were disrupted by sonication for a total of 4 min and the lysate was cleared of debris by centrifugation at 13 000 rpm (19 685 g) for 30 min at 4°C. The supernatant was filtered using a 0.45 μm syringe filter, loaded onto a 5 ml HiTrap chelating column charged with NiS0_4_, and then washed with nickel load buffer. Proteins were eluted with an imidazole gradient from 30 mM to 1 M. Pooled fractions were dialyzed overnight into TCBg50 buffer (20 mM Tris pH 8.0, 50 mM NaCl, 1 mM EDTA, 5% glycerol, 1 mM DTT) and further purified by anion exchange and size exclusion chromatography (SEC), using a 5 ml HiTrap Q HP column and a Superdex 75 16/600 pg column respectively. Proteins were exchanged into a final buffer of 20 mM HEPES pH 7.5, 150 mM KCl, 5 mM MgCl_2_, and 1 mM DTT during SEC and concentrated to 10–40 mg/ml.

Active site mutations were introduced via Quikchange and mutants were expressed and purified in the same manner as wildtype.

### Inductively coupled plasma atomic emission spectroscopy (ICP-AES)

Bp^CTR^ and Xcc^CTR^ were cloned into the expression vector pASK-IBA3C, introducing a C-terminal Strep-II tag. Strep-tagged CTR constructs were transformed into BL21(DE3) cells, grown at 37°C in Terrific Broth to an OD_600_ of 0.7–0.9, and then induced with 0.3 mM IPTG overnight at 19°C. Cells were harvested and washed in Strep buffer (100 mM Tris–HCl pH 8.0, 500 mM NaCl, 5 mM β-mercaptoethanol). Pellets were resuspended in 50 ml of Strep buffer supplemented with 3 mg DNAse, 2 mM MgCl_2_, 10 mM PMSF, a Roche complete protease inhibitor cocktail tablet, and 1 mg/ml lysozyme. Following a 10 min incubation at 4°C, the cells were sonicated and cleared via centrifugation. The supernatant was filtered, loaded onto a 5 ml StrepTrap column, and washed with Strep buffer. The protein was eluted with Strep buffer supplemented with 2.5 mM *d*-desthiobiotin. The protein was pooled, concentrated, and injected onto a Superdex 75 10/300 GL column. Bp^CTR^ and Xcc^CTR^ were exchanged into a final buffer of 20 mM HEPES pH 7.5 and 50 mM NaCl, which had been first passed through Chelex 100 resin to remove contaminating divalent cations. The final protein sample was concentrated to ∼10 mg/ml. Approximately 500 μl of each protein sample was dried under vacuum and resuspended in 10 ml of 2% nitric acid. Samples were analyzed with an iCAP 6000 ICP-ES, Thermo. Measurements were done in triplicate. The determined milliequivalents of metal per protein molecule are listed in Supplementary Table S1.

### Crystallization, X-ray data collection, and structure determination

Xcc^CTR^ was crystallized by sitting drop vapor diffusion in 0.1 M Bis Tris Propane pH 7.0-8.0, 11–26% PEG 3350, and 0.15–0.2 M sodium iodide with a drop size of 2 μl and reservoir volume of 65 μl. Crystals appeared within seven days at 20°C. Native crystals grown in the presence of iodide were stabilized with 0.25 M NDSB-195 or soaked with either 10 mM potassium pentaiodoplatinum IV for 45 min or 10 mM ethylmercury chloride for 45 min. All samples were cryoprotected by transfer to 100% paratone-N, allowing all mother liquor to exit the crystal prior to freezing in liquid nitrogen. Crystals were of the space group *P*4_3_ with unit cell dimensions *a* = *b* = 65.4 Å, *c* = 63.8 Å and α = β = γ = 90°. Crystals were screened and optimized at the MacCHESS F1 beamline at Cornell University and X-ray diffraction data was collected remotely on the tuneable NE-CAT 24-ID-C beamline at the Advanced Photon Source. Single-wavelength anomalous diffraction (SAD) (30) datasets were collected on a Dectris Pilatus 6MF pixel array detector at 100 K for the platinum, mercury, and iodide derivatives at the energies of 12 300, 11 570, and 7500 eV, respectively. Datasets were integrated and scaled with XDS (31) and Aimless (32) via the RAPD pipeline. Heavy atom sites were located using SHELX (33) and phasing, density modification, and initial model building was carried out using the Autobuild routine of PHENIX (34). Initial figures of merit following density modification was 0.62 for Xcc^CTR^ Pt, 0.64 for Xcc^CTR^ Hg, and 0.504 for Xcc^CTR^ I. Further model building and refinement was carried out in COOT (35) and PHENIX (34) respectively. The final models were refined to the following resolutions and *R*_work_/*R*_free_: Xcc^CTR^ Pt, 1.86 Å, 0.212/0.238; Xcc^CTR^ Hg, 1.95 Å, 0.201/0.241; Xcc^CTR^ I, 2.30 Å, 0.215/0.275 (Supplementary Table S2).

Bp^CTR^ was crystallized by sitting drop vapor diffusion in 0.1 M HEPES pH 7.5, 0.23 M MgCl_2_, 30% PEG 400 and 0.001 M glutathione with a drop size of 1 μl and reservoir volume of 65 μl. Crystals appeared within 2–3 days at 20°C. Samples were cryoprotected by transfer to 100% paratone-N, allowing all mother liquor to exit the crystal prior to freezing in liquid nitrogen. Crystals were of the space group *C*222_1_ with unit cell dimensions *a* = 83.256 Å, *b* = 105.669, *c* = 123.764 and α = β = γ = 90°. X-ray diffraction data were collected remotely on the NE-CAT 24-ID-C beamline at the Advanced Photon Source at 100 K on a Dectris Pilatus 6MF pixel array detector. The dataset was integrated and scaled using XDS and Aimless via the RAPD pipeline. The structure was solved by molecular replacement in PHASER (36) using the refined Xcc^CTR^ Pt-soaked structure as the search model. Two molecules were found in the asymmetric unit. Model building and refinement were carried out in COOT (35) and PHENIX (34) respectively. The final model was refined to 2.24 Å resolution with an *R*_work_/*R*_free_ of 0.213/0.260 (Supplementary Table S2). The model also contained difference density peaks in the active site that were modeled as two magnesium ions based on the geometry and the components of the crystallization condition.

All structural renderings were generated using Pymol (Schrodinger) and surface electrostatics were calculated using APBS (37). Conservation based coloring was generated using the ConSurf server (38).

### Size-exclusion chromatography coupled to multi-angle light scattering (SEC-MALS)

Purified Bp^FL^ (4 mg/ml), Xcc^FL^ (4 mg/ml), Bp^CTR^ (6 mg/ml) and Xcc^CTR^ (6 mg/ml) were subjected to SEC using a Superdex 200 10/300 gl (GE) column equilibrated in SEC-MALS buffer (20 mM HEPES pH7.5, 150 mM NaCl, 5 mM MgCl_2_ , 1 mM DTT). The gel filtration column was coupled to a static 18-angle light scattering detector (DAWN HELEOS-II) and a refractive index detector (Optilab T-rEX) (Wyatt Technology). Data were collected continuously at a flow rate of 0.5 ml/min. Data analysis was performed using the program Astra VI. Monomeric BSA (6.0 mg/ml) (Sigma) was used for normalization of the light scattering detectors and data quality control.

### DNA cleavage assays

100 ng of lambda DNA or pUC19 plasmid DNA was mixed with 8 μM protein to a final volume of 20 μl in DNA cleavage buffer (20 mM Tris-OAc pH 7.9, 50 mM KCl, 0.1 mg/ml BSA,10 mM divalent metal). Reactions were incubated at 37°C for 60 min and quenched with 5 μl of 0.5 M EDTA pH 8.0. Samples were analyzed via native agarose electrophoresis. DNA degradation was quantified using BioRad Image Lab software and assessed by measuring the amount of ethidium bromide signal in each lane and comparing it to the protein-free DNA sample. Bar graphs represent the average of three independent trials with error bars representing the standard error of the mean. Mutant constructs were assayed in the presence of 10 mM MgCl_2_ and 1 mM CaCl_2_ based on ICP-AES and metal titration results.

### Exonuclease assays

The following DNA oligonucleotides for exonuclease assays were synthesized commercially by Integrated DNA Technologies (IDT):

Exo_US (5′ or 3′ labeled with 6-carboxyfluorescein, 6-FAM)

5′-CTCACTGGTGCTAGGCAACGTTGAAGTGATCGTACGCGGA-3′

Exo_WT_LS

5′-TCCGCGTACGATCACTTCAACGTTGCCTAGCACCAGTGAG-3′

Exo_GT_LS

5′-TCCGCGTACGATCACTTCAACGTTGCCTGGCACCAGTGAG-3′

Lyophilized single-stranded oligonucleotides were resuspended to 1 mM in 10 mM Tris–HCl and 1 mM EDTA and stored at −20°C until needed. Duplex substrates were prepared by heating equimolar concentrations of complementary strands (denoted with suffixes ‘us’ and ‘ls’ indicating upper and lower strands) to 95°C for 15 min followed by cooling to room temperature overnight. Four substrates were prepared: two wildtype substrates (5′ or 3′ 6FAM-labeled Exo_US each with Exo_WT_LS) and two G:T mismatched substrates (5′ or 3′ 6FAM-labeled Exo_US each Exo_GT_LS). For each substrate, a 150 μl reaction containing 8 μM protein and 75 pmol of labeled double stranded DNA was prepared in exonuclease buffer (20 mM Tris-OAc pH 7.9, 50 mM K-OAc, 0.1 mg/ml BSA, 10 mM MgCl_2_ ,1 mM CaCl_2_) and incubated at 37°C. 20 μl aliquots were taken at the indicated time points and quenched with 3× loading buffer (80% formamide and 1X TBE). Samples were analyzed by a denaturing (8 M urea) 14% polyacrylamide gel and visualized using Bio-Rad ChemiDoc XRS+.

## RESULTS

### Identification and classification of OLD homologs

Recombinant expression of P2 OLD produced unstable protein that aggregated and/or precipitated, regardless of the tag or conditions employed. We therefore searched the KEGG database (39) to identify OLD homologs more suitable for structural and biochemical characterization. The initial search was carried out using the *E. coli* K12 MG1665 OLD homolog (KEGG ID eco:b0876), which is annotated as the uncharacterized protein YbjD and is 18% identical and 35% similar to P2 OLD. These efforts yielded 833 homologs distributed across numerous kingdoms but absent in eukaryotes (Supplementary Table S3). A further search of mapped plasmid genomes available in the Integrated Microbial Genomes database (40) yielded four additional OLD homologs. We then examined the genetic context of each *old* gene, as inspection of gene neighborhoods has been shown to elucidate unanticipated genetic connections and facilitate new functional predictions (41). Our analyses show that *old* genes segregate into two primary classes (Supplementary Table S3). Class 1 OLD family members (542/837)—including P2 phage, *Escherichia coli*, and *Salmonella typhimurium*—exist as single, isolated genes (Supplementary Figure S1A). Class 2 OLD homologs (295/837) appear in tandem with a UvrD/PcrA/Rep-like helicase (Supplementary Figure S1B), often as an overlapping reading frame. UvrD, PcrA, and Rep are non-hexameric, superfamily 1A helicases that translocate with a 3′-5′ polarity and play essential roles in DNA replication, recombination, and repair (42,43). Both classes retain the conserved motifs characteristic of ATPase and Toprim domains, though Class 1 proteins are on average ∼50 amino acids shorter. Each class appears in a number of different phyla, with examples present in both Gram positive and Gram negative bacteria, archaea, and bacteriophage viruses.

**Figure 1.**
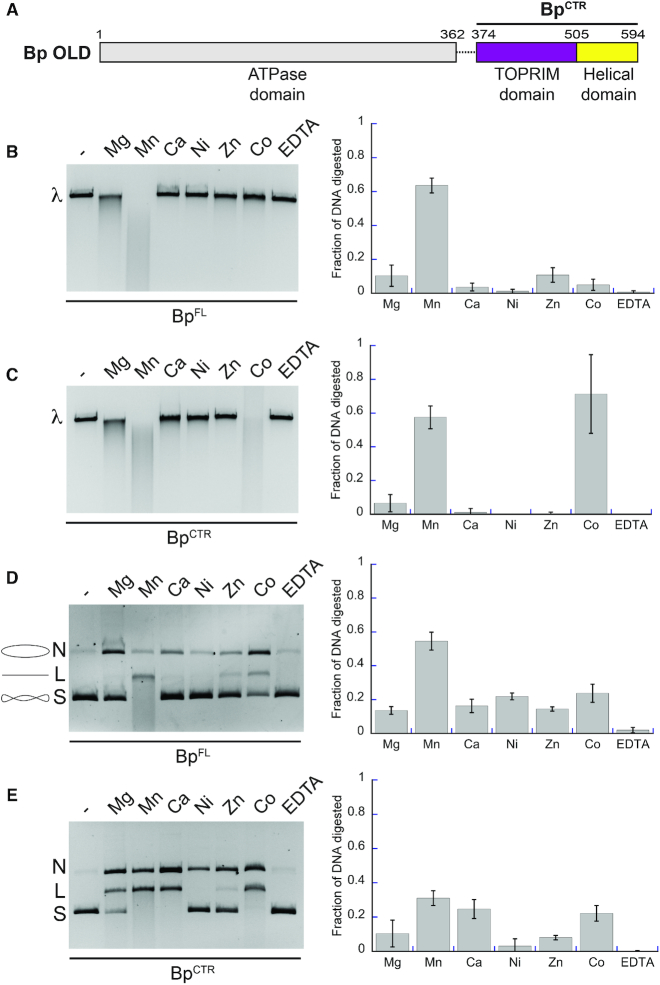
Bp OLD exhibits metal-dependent nuclease activity. (**A**) Domain architecture of Bp OLD. Boundaries of the CTR construct is labeled. (**B**, **C**). Metal-dependent nuclease activity of Bp^FL^ (B) and Bp^CTR^ (C) on linear λ DNA. ‘λ’ denotes the position of uncut linear DNA. (**D**, **E**) Metal-dependent nuclease activity of Bp^FL^ (D) and Bp^CTR^ (E) on supercoiled pUC19 DNA. ‘N’, ‘L’, and ‘S’ denote the positions of ‘nicked’, ‘linearized’, and ‘supercoiled’ DNA respectively. Cartoons of these respective products are illustrated in D next to their label. For B–E, lanes labeled with dashed lines indicate samples with no metal added. DNA degradation was quantified using BioRad Image Lab software as described in the Materials and Methods. Bar graphs represent the average of three independent experiments with error bars representing the standard error of the mean.

A subset of *old* genes (107/837) exist in species-specific operons (Supplementary Table S3). Neighboring genes within these operons contribute to numerous biological functions including bacterial defense, DNA replication and repair, transcriptional regulation, membrane transport, biosynthesis, metabolism, and signaling (Supplementary Table S3).

We selected numerous candidates from each class for expression studies. Like P2 OLD, most Class 1 homologs behaved poorly during purification. Class 2 homologs, in contrast, were intrinsically more stable and generally provided greater yields of soluble, monodispersed protein. Specifically, the Class2 OLD homologs from *B. pseudomallei* and *X. campestris* p.v. *campestris* could be purified to homogeneity (Figure 1A, Supplementary Figure S1C and D) and concentrated to greater than 10 mg/ml without appreciable aggregation or precipitation. Size exclusion chromatography coupled to multi-angle light scattering (SEC-MALS) indicates that Xcc OLD forms stable tetramers in solution while Bp OLD (Bp^FL^) exists in equilibrium between dimers and tetramers (Supplementary Figure S1E). In contrast, truncated constructs containing the C-terminal region of each homolog (Xcc^CTR^, Bp^CTR^; Figure 1A and Supplementary Figure S1C and D) were each monomeric by SEC-MALS analysis (Supplementary Figure S1F).

### Class 2 OLD proteins exhibit metal-dependent DNA cleavage *in vitro*

Metal-dependent nicking and cleavage of nucleic acid substrates is a hallmark of Toprim domain-containing proteins (44). To verify that purified Class 2 OLD proteins share a similar activity *in vitro*, we incubated Bp^FL^ with linearized λ phage DNA in the presence of different divalent cations (Figure 1B). Cleavage activity was quantified by measuring the ethidium bromide signal in each lane and calculating the fraction of DNA digested relative to the untreated substrate, which increased under conditions that promote nuclease function. Bp^FL^ exhibits cleavage in the presence of Mg^2+^, degrading approximately 10% of the substrate within an hour. Activity is enhanced in the presence of Mn^2+^, where 60% of the DNA substrate is degraded (Figure 1B). We also observe weak activity in presence of Zn^2+^ and Co^2+^. Bp^CTR^ similarly shows cleavage with Mg^2^ (5% degradation) and Mn^2+^ (60%), though it is also highly active in the presence of Co^2+^ (70%) (Figure 1C). Given that Co^2+^ only stimulates activity in Bp^CTR^, we suspect that this is a construct-specific artifact rather than a general feature of the OLD nucleases. Xcc^FL^ and Xcc^CTR^ similarly can degrade DNA with Mg^2+^and Mn^2+^ but also are partially active in Zn^2+^ (Supplementary Figure S2A and B). These data indicate that the critical catalytic resides associated with nuclease function reside in the C-terminal half of OLD proteins and that the N-terminal region containing the ATPase domain is not required for DNA binding or nuclease activity.

We next assessed the ability of Bp OLD to nick and cleave circular plasmids. Bp^FL^ was mixed with supercoiled pUC19 DNA (S) in the presence of different divalent metals and activity was evaluated by the appearance of slower migrating bands as the substrate was nicked (N) and linearized (L) by the enzyme (Figure 1D). Bp^FL^ shows weak nicking activity with all metals as compared to the DNA alone and EDTA controls (Figure 1D), with Mg^2+^, Mn^2+^ and Co^2+^ again eliciting the strongest nicking effects. Under these conditions, only Mn^2+^ promotes processive cleavage, degrading 55% of the circular substrate (Figure 1D). Bp^CTR^ shows pronounced nicking activity in the presence of every metal tested, with some processive cleavage stimulated by Mn^2+^ (31% degraded), Co^2+^ (22% degraded), and Ca^2+^ (24% degraded) (Figure 1E). Xcc^FL^ and Xcc^CTR^ show the strongest nicking and cleavage activities on supercoiled DNA with Mg^2+^, Mn^2+^ and Zn^2+^, though XccCTR appears to be able to nick Ca^2+^ as well (Supplementary Figure S2C and D). We note that the extent of cleavage in Xcc is less than Bp overall, suggesting it is a less efficient nuclease.

Given the variation in nuclease function we observed for Bp and Xcc OLD with different metals *in vitro*, we sought to identify which metals are preferentially bound to the CTR constructs *in vivo* using inductively coupled plasma atomic emission spectroscopy (ICP-AES). This technique can measure the type and amount of metal in a given sample with high accuracy (45). Bp^CTR^ and Xcc^CTR^ constructs were purified using Strep-II tags to avoid any confounding results arising from coincidental metal binding to a His tag. ICP-AES showed calcium to be the most abundant metal associated with both Bp^CTR^ (79.35 mEq) and Xcc^CTR^ (100.27 mEq), followed by magnesium (18.38 and 11.39 mEq, respectively), and then by zinc and nickel (Supplementary Table S1). Sparing amounts of cobalt were detected in the Bp^CTR^ sample, suggesting it is not as physiologically relevant. No manganese was found in either sample (Supplementary Table S1). Given the unexpected presence of calcium, we tested if it may play a role in modulating nuclease activity. Presence of Ca^2+^ alone does not promote robust nuclease activity on linear or supercoiled DNA substrates; however, a combination of Ca^2+^ and Mg^2+^ enhances the activities of both Bp^CTR^ and Xcc^CTR^ above either metal alone (Supplementary Figure S3A and B). Nuclease activity is most stimulated with Mg^2+^ in excess and Ca^2+^ between 1 and 2 mM. Under these optimal conditions, the activity of both Bp^CTR^ and Xcc^CTR^ is stimulated more than 10-fold on linear DNA compared to Ca^2+^ or Mg^2+^ alone. Degradation of circular DNA was also enhanced 4–5-fold for Bp^CTR^ and Xcc^CTR^. Addition of ATP had no appreciable effect on Bp^FL^ cleavage of either substrate (linear versus supercoiled DNA) in the presence of optimal concentrations of Ca^2+^ and Mg^2+^ (Supplementary Figure S3C), further underscoring the notion that the CTR mediates the DNA binding and nuclease functions. Taken together, our results imply that Ca^2+^ acts as an important modulator of OLD family nuclease activity and can potentiate the catalytic effects of these enzymes in the presence of Mg^2+^.

The robust degradation of linear substrates we observe does not explicitly distinguish between exo- and endonuclease activities. To test the exonuclease function and directionality, we incubated Bp^CTR^ with a 40 bp double stranded DNA substrate labeled on the 5′ or 3′ end with 6-carboxyfluorescein (6-FAM). Bp^CTR^ degrades the 3′-labeled substrate in a stepwise manner (Supplementary Figure S4A) while no intermediates or laddering is observed on the 5′-labeled substrate (Supplementary Figure S4B). These findings indicate Bp^CTR^ can act as an exonuclease that digests DNA in the 5′-3′ direction as well as an endonuclease that can act on supercoiled, circular DNA substrates.

### Overall structures of the Xcc and Bp OLD C-terminal regions

**Figure 2.**
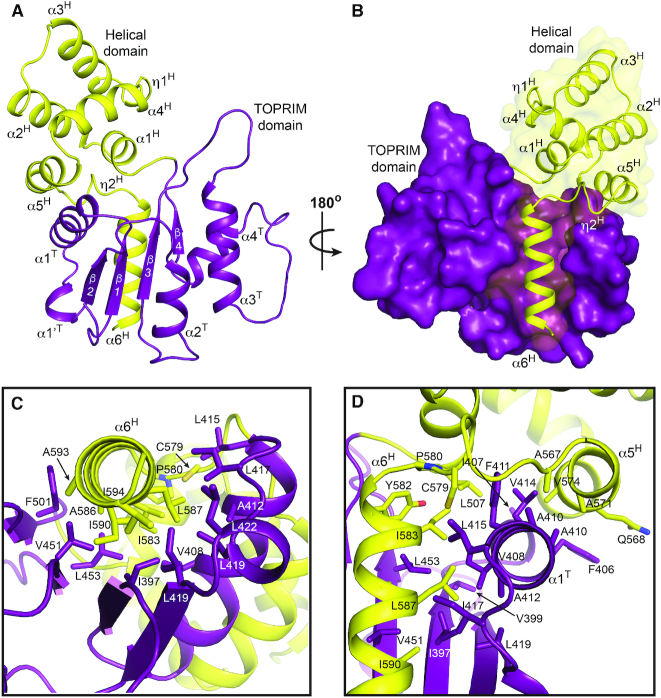
Structure of Bp**^CTR^**. (**A**) Structure of Bp^CTR^. Toprim, and helical domains are colored purple and yellow respectively. (**B**) Surface representation of domain interactions. (**C, D**) Structural interactions between Toprim and helical domains. Side chains involved in stabilizing hydrophobic interactions shown as sticks are labelled.

Although full-length Xcc and Bp OLD proteins crystallize in the presence of different adenine nucleotides, diffraction rarely exceeded ∼4 Å and interpretable electron density maps could not be obtained owing to severe radiation damage. Isomorphous crystals were never observed for any condition screened thereby preventing merging of data. The truncated Xcc^CTR^ construct, in contrast, yielded crystals that routinely diffracted beyond 2 Å and three independent structures were solved using SAD datasets from platinum, mercury, and iodide derivatives (Supplementary Table S2, Supplementary Figures S5 and S6A). These models show strong agreement with an overall RMSD of 0.42–0.44 Å and display only slight deviations at the N-terminus near the mercury-binding site and within a flexible loop containing two adjacent glycines (G479 and G480) (Supplementary Figure S6A). Residues 374–387 and 458–463 are disordered in each structure, though present in the purified construct. Crystals of the analogous Bp^CTR^ construct diffracted to a slightly lower resolution (2.2–2.3 Å) but produced a more complete structural model (residues 390–594; Figure 2).

Bp^CTR^ contains two domains (Figure 2A): a Toprim domain (residues 390–504, purple) and a unique helical domain (residues 505–594, yellow) consisting of a five-helix orthogonal bundle and an additional C-terminal amphipathic helix (α6^H^). α6^H^ extends into a groove along one face of the Toprim's central β-sheet, forming extensive hydrophobic interactions (Figure 2B and C). Helix α5^H^ and the upper portion of α6^H^, along with the connecting loop, wrap around the hydrophobic helix α1^T^ of the Toprim domain to stabilize the structure further. The contributing hydrophobic side chains are largely conserved among Class 2 OLD proteins (Supplementary Figure S7) and together bury a total surface area of 1341Å^2^. Similar interactions are observed between the domains in the Xcc^CTR^ (Supplementary Figure S5). Attempts to express Bp^CTR^ and Xcc^CTR^ constructs lacking α6^H^ were unsuccessful as deletion of α6^H^ rendered the proteins insoluble. This likely reflects the critical stabilizing interactions provided by conserved residues along the α6^H^-β-sheet interface and the exposure of a large hydrophobic surface if this helix is absent.

**Figure 3.**
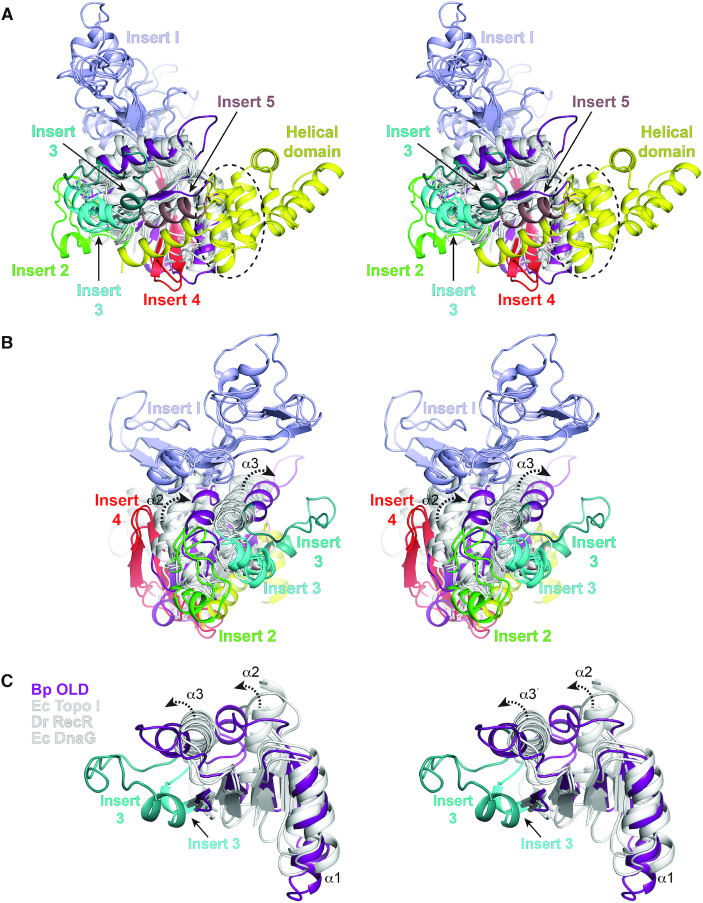
Topological differences in present in Class 2 OLD Toprim domains. (**A**, **B**) Structural superposition of Toprim domains. Side (A) and end (B, rotated 90°) views are shown in stereo. Structural alignment includes the following: *Geobacillus kaustophilus* conserved hypothetical protein (PDB: 2FCJ), *Escherichia coli* DnaG primase (PDB: 3B39), *Aquifex aeolicus* putative ribonuclease M5 (PDB: 1T6T), *Deinococcus radiodurans* RecR (PDB: 1VVD), *Thermotoga maritima* Topoisomerase I (PDB: 2GAJ), *Escherichia coli* Topoisomerase I (PDB: 1MW9), *Homo sapiens* Topoisomerase IIB (PDB: 3QX3), *Streptococcus pneumoniae* IV topoisomerase (PDB: 4I3H), *Escherichia coli* Topoisomerase III (PDB: 2054), *Archaeoglobus fulgidus* reverse gyrase (PDB: 1GKU), and *Thermotoga maritima* reverse gyrase (PDB: 4DDU). Toprim cores are colored white with Inserts 1–5 individually labeled (see Supplementary Figures S8 and S9). Bp Toprim and helical domains are colored purple and yellow respectively. Dashed circle in (A) denotes the position of the glutamate helix. (**C**) Stereo view of α2 and α3 helical shifts (dashed arrows) in Bp OLD Toprim core relative to the Toprim central β-sheet. Central segments of *Escherichia coli* (Ec) Topoisomerase I, *Deinococcus radiodurans* (Dr) RecR and *Escherichia coli* DnaG are shown for comparison. Alternative position of Insert 2 in Bp OLD is also shown.

Many Toprim family members contain individual structural inserts into the core Toprim fold (Figure 3, Supplementary Figures S8 and S9). These include an insertion of variable size and structure between β2 and α2 in topoisomerases, gyrases, and RecR (Insert 1, light blue), short helical insertions between α2 and β3 (Insert 2 green) and α3 and β4 (Insert 3, cyan) in topoisomerases, a two-stranded β-hairpin added between α1 and β1 that extends the central β sheet in gyrases and topoisomerase III (Insert 4, red), and an α helix following the shortened β4 in the putative RNase M5 from *Aquifex aeolicus* (Insert 5, brown). Bp and Xcc OLD lack most of these embellishments but contain an Insert 3 helix (Figure 3, Supplementary Figures S8 and S9, Teal). Class 2 OLD proteins show sequence variability across this insert region (Supplementary Figure S7). Significantly, structural superposition reveals a shift of the Toprim α2 and α3 helices in OLD proteins relative to all other Toprim family members (Figure 3B and C, Supplementary Figure S8) while the rest of the core fold is largely unchanged (Figure 3C, Supplementary Figure S8). The position of these helices is consistent between the Bp^CTR^ and Xcc^CTR^ structures, arguing it is an intrinsic topological feature and not simply due to crystal packing. This comparison also shows that the OLD helical domain is distinctly separated from all other inserts, localized on the opposite side of the Toprim fold (Figure 3A). We do note that DnaG primases and the putative *A. aeolicus* RNase M5 contain a helix that structurally aligns with the α1^H^ helix of the OLD helical domain (Figure 3A, dashed circle).

**Figure 4.**
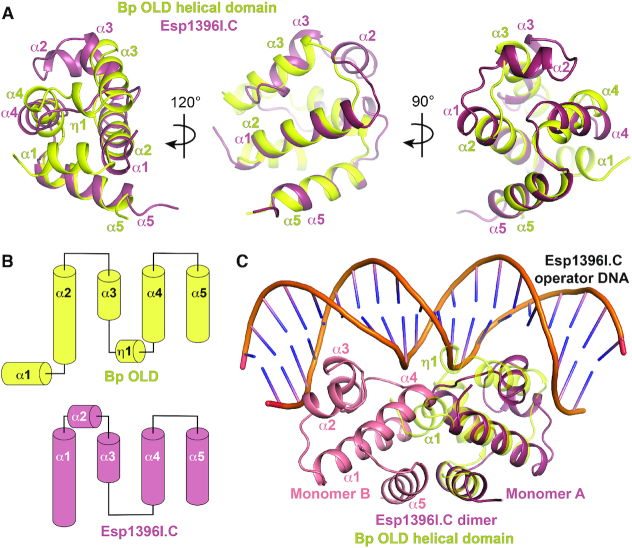
Helical domain in Class 2 OLD proteins shares structural homology with R–M system controller proteins. (**A**) Structural superposition of Bp OLD helical domain (yellow) with Esp1396I.C controller protein (raspberry; PDB: 3CLC). (**B**) Topology diagrams of Bp OLD helical domain and Esp1361.C monomer. (**C**) Bp OLD architecture precludes dimerization as in controller proteins and suggests an alternate mode of DNA binding.

The helical domain shares structural homology with bacterial controller (C) proteins from restriction–modification (R–M) systems (top hit from the DALI server (46): C.Esp1396I, *Z* score: 5.1, RMSD 2.5 Å) (Figure 4). C proteins act as transcriptional regulators that tune the expression of R-M methyltransferase and restriction genes to ensure that site-specific nuclease activity is delayed until after a bacterial genome is protected by methylation (47). Crystallographic studies have shown that these proteins are dimeric and α-helical, with each monomer containing a helix-turn-helix motif (48). Structural superposition aligns Bp helices α2, α3, α5 and α6 with α1, α3, α4, and α5 of C.Esp1396I (Figure 4A and B). Bp OLD lacks a helix corresponding to C.Esp1396I α2 and contains two additional helices (α1 and α4) that localize to the opposite side of the molecule (Figure 4A and B). C protein dimers bind DNA operator sites cooperatively to exert concentration-dependent switching of promoter activation and repression (47,49). In this arrangement, α4 facilitates dimerization while α2 and α3 associate with DNA (Figure 4C). The Bp α1 and α4 helices would sterically block dimerization and DNA interactions respectively, thus preventing OLD proteins from adopting a similar configuration.

### Bp OLD active site suggests a two-metal catalysis mechanism

**Figure 5.**
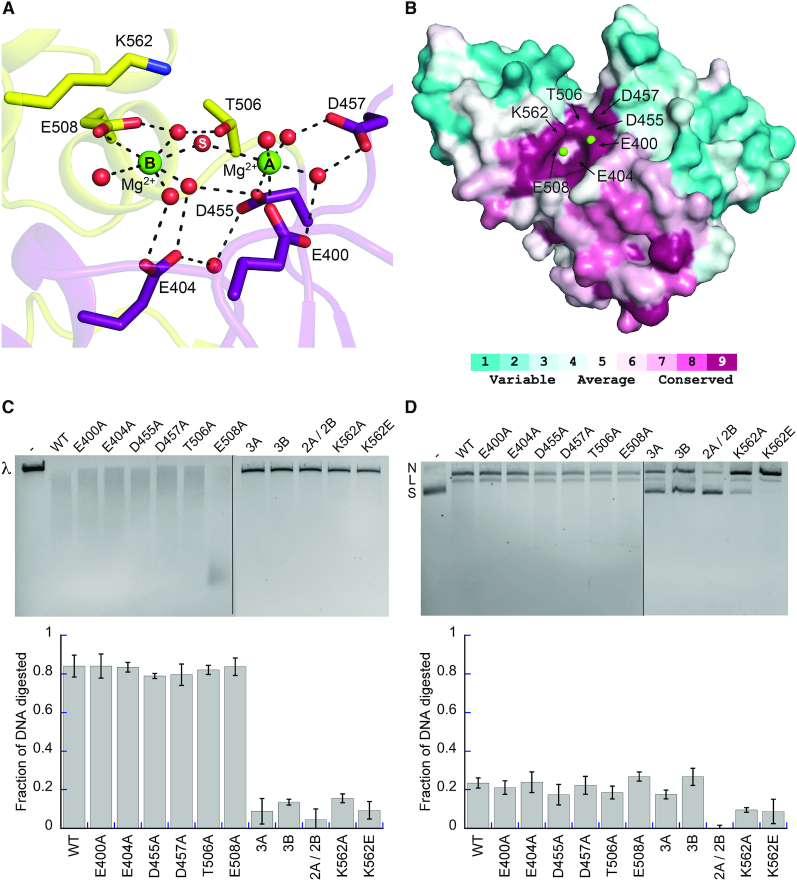
Bp OLD active site geometry is consistent with two-metal catalysis. (**A**) Metal coordinating residues in the Bp OLD active site. Ordered magnesium ions (green) and water molecules (red) are shown as spheres. Dashed lines indicate hydrogen bonds. (**B**) Conservation of active site residues. Coloring generated using the ConSurf server (38) and the alignment in Supplementary Figure S7. (**C**) Cleavage activities of Bp^CTR^ active site mutants on linear λ DNA. 3A denotes triple mutant in the metal A binding site (E400A/D455A/D457A), 3B signifies triple mutant in the metal B binding site (E404A/T506A/E508A), 2A/2B contains mutations in both metal-binding sites (D455A/D457A/T506A/E508A). (**D**) Nicking and cleavage activities of Bp^CTR^ mutants on supercoiled pUC19 DNA. ‘N’, ‘L’ and ‘S’ denote the positions of ‘nicked’, ‘linearized’, and ‘supercoiled’ DNA respectively. All cleavage assays were performed with 10 mM MgCl_2_ and 1 mM CaCl_2_. Lanes labeled with dashes in C and D indicate substrate alone controls without protein added. DNA degradation was quantified using BioRad Image Lab software as described in the Materials and Methods as described in the Materials and Methods. Bar graphs show the average of three independent experiments with error bars representing the standard error of the mean.

The Xcc^CTR^ derivative structures contain nothing in their active sites. Bp^CTR^ crystallized in a different space group thereby permitting the helical domain to rotate and scrunch closer to the Toprim domain (Supplementary Figure S6B). Consequently, T506 and E508 shift 1.2 and 1.5 Å toward the active site respectively, which facilitates the binding of two magnesium ions in a geometry consistent with two metal catalysis (Figure 5A, Supplementary Figure S6C). Each magnesium is octahedrally coordinated with a water molecule bridging the two metals where the scissile phosphate would normally sit (Figure 5A). The metals are spaced 4.9 Å apart, suggesting they may move closer together once DNA is engaged. The conserved Toprim glutamate (E400) and the first aspartate of the DxD motif (D455) each provide a ligand to the first magnesium (metal A). The second DxD aspartate (D457) hydrogen bonds with two waters that form two additional metal A ligands. E508, located in αH1 of the helical domain, directly coordinates the second magnesium (metal B), while E404 and T506 stabilize additional metal B waters. These side chains are absolutely conserved in Class 2 OLD nucleases (Figure 5B, Supplementary Figure S7). Individual substitutions of metal A ligands (E400A, D455A, D457A) and metal B ligands (E404A, T506A, E508A) yielded no discernible effects on cleavage activity (Figure 5C and D). Thus, combinations of mutations (3A, E400A/D455A/D457A; 3B, E404A/T506A/E508A) were generated. Mutant combinations of either the metal A or the metal B interacting residues together completely abolish Bp OLD nuclease activity on linear DNA substrates *in vitro* (Figure 5C). These substitutions impair the processive degradation of circular plasmids in the presence of Mg^2+^ and Ca^2+^, though some nicking activity is still retained (Figure 5D). Simultaneous mutation of both metal A and metal B sites together (2A/2B, D455A/D457A/T506A/E508A) eliminates processive cleavage and significantly reduces nicking activity relative to the 3A and 3B substitutions (Figure 5C and D). This suggests that a single metal in either site can facilitate nicking but both sites are required for processive cleavage and degradation.

We also identify a conserved lysine residue in α5^H^ (K562) that extends toward the active site (Figure 5A and B, Supplementary Figure S7), separated from metal A by 5.5 Å and from metal B by 3.8 Å. K562A and K562E mutations similarly impair processive nuclease activity without perturbing direct interactions with either magnesium (Figure 5C and D). K562A and K562E mutations, however, retain the ability to nick DNA (Figure 5D), similar to the perturbation of the individual metal A and B binding sites. Together these data define the key catalytic machinery of Class 2 OLD nucleases and support a two-metal catalysis mechanism for processive nuclease activity.

**Figure 6.**
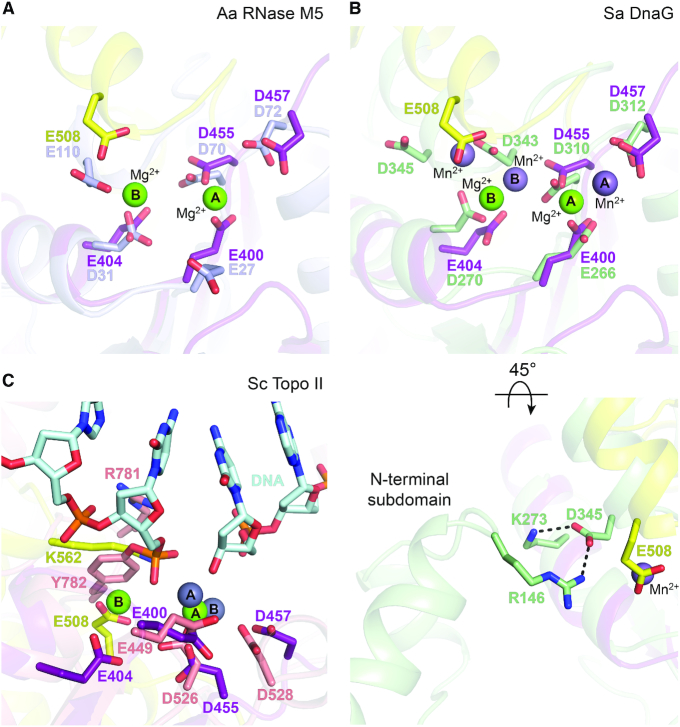
Conservation of active site elements among OLD nucleases, RNase M5 maturases, and DnaG primases. Comparison of Bp^CTR^ active site (Toprim domain, purple; helical domain, yellow) with different Toprim family members. Critical side chains in each protein are labeled and the bound metal A and metal B magnesiums are shown as green spheres. (**A**) Superposition with the putative RNase M5 from *Aquifex aeolicus* (light blue, PDB: 1T6T). (**B**) Superposition with DnaG primase from *Staphylococcus aureus* (light green, PDB: 4EE1). Bound manganese ions in the Sa DnaG structure are shown as light purple spheres. Lower panel is rotated and recentered to illustrate interactions between N-terminal subdomain and the Toprim within the Sa DnaG RNA polymerase core. Dashed lines indicate hydrogen bonds. (**C**) Superposition with *Saccharomyces cerevisiae* topoisomerase II (salmon, PDB: 3L4K). Bound zinc ions in the Sc Topo II structure are shown as gray spheres. Covalently-linked DNA substrate is colored cyan.

The organization of the Bp OLD active site is structurally conserved in RNase M5 enzymes and DnaG primases (Figure 6, Supplementary Figure S9). Along with the invariant Toprim glutamate and conserved DxD aspartates, D31 and E110 in the *A. aeolicus* RNase M5 homolog spatially align with E404 and E508 in Bp OLD (Figure 6A). E110 localizes to a C-terminal helix that superimposes with α1^H^ of the Bp OLD helical domain (Figure 3A, dashed circle; Supplementary Figure S9, inset). DnaG primases contain a similar set of catalytic machinery (16). The analogous C-terminal acidic residue of the DnaG Toprim (D345 in *Staphylococcus aureus*), however, is directed away from the active site via interaction with a conserved arginine residue (R146 in *Staphylococcus aureus*) in the adjacent N-terminal subdomain of the RNA polymerase core (Figure 6B). As a result, a third metal (metal C) binds in the position occupied by E508 in Bp OLD, coordinated by a conserved aspartate residue immediately upstream (D343 in the *Staphylococcus aureus*) (20) (Figure 6B). The arrangement of metals relative to the core catalytic side chains in these enzymes is distinct from the coordination observed in topoisomerases, where metal B is positioned closer to the DxD motif in the absence of additional acidic residues (Figure 6C). Metal B and the catalytic lysine in OLD proteins occupy the same position as the catalytic tyrosine that forms a covalent linkage with DNA in toposiomerases (Y782 in *Saccharomyces cerevisiae* topoisomerase II) (50). These differences highlight the evolutionary fine tuning of the Toprim scaffold for unique biological functions.

### Structural model for DNA binding

**Figure 7.**
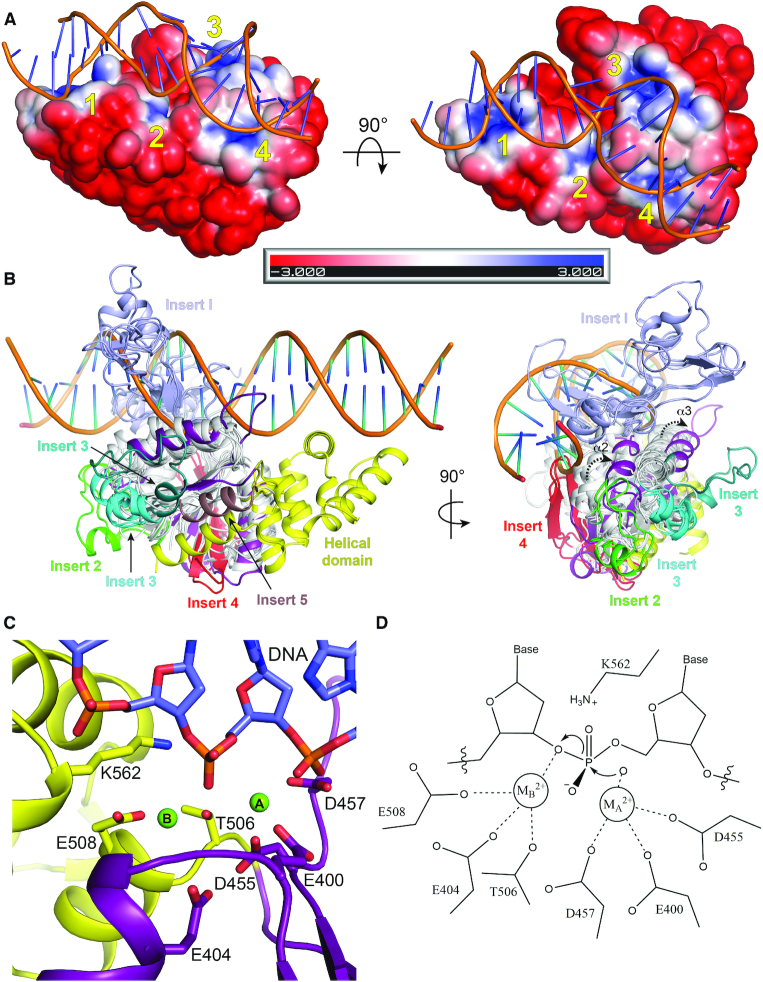
Helical domain orients DNA binding in Class 2 OLD nucleases. (**A**) Electrostatic surface of Bp^CTR^ with modeled DNA (G:T mismatched substrate taken from PDB: 3K0S). Electrostatic potential calculated with APBS (37). Scale indicates coloring of the potential from –3*K*_b_*T*/e_c_ to +3*K*_b_T/e_c_. The four basic patches around the active site cleft are numbered in yellow. (**B**) Side and end views of Toprim family superposition with modeled DNA substrate bound to Bp OLD^CTR^. Toprim cores are colored white with Inserts 1–5 individually labeled. Bp Toprim and helical domains are colored purple and yellow respectively. (**C**) Arrangement of Bp^CTR^ active site with modeled DNA. (**D**) Proposed two-metal catalysis mechanism for Class 2 OLD nuclease cleavage.

Our attempts to co-crystallize OLD proteins with nucleic acids have thus far been unsuccessful. The robust nuclease activity exhibited by Bp^CTR^ suggests that this fragment alone can associate with DNA in a manner that is competent for cleavage. We therefore computationally modeled DNA onto the Bp^CTR^ structure to gain insight into how OLD nucleases interact with their substrates. Calculation of surface electrostatics identifies four basic patches on one face of Bp^CTR^ that flank a small cleft containing the active site (Figure 7A). Patch 1 lies between α3^H^ and α4^H^ in the helical domain, formed by R552, K555 and R559 (Supplementary Figure S10A). The catalytic K562 lysine on α5^H^ constitutes patch 2. As noted above, this extends into the active site and has a direct role in nuclease activity (Figure 5C and D). Patch 3 localizes along α3^T^ in Toprim domain, comprised of R467 and K468, while patch 4 contains R405, which extends from α1^T^ toward β2 (Supplementary Figure S10A). Modeled B form DNA can bind patches 1 and 2 and part of patch 3, but sterically clashes with the protein beyond the active site cleft (Supplementary Figure S10B). In contrast, we obtain a near optimal fit with a bent DNA substrate taken from a co-crystal structure of the bacterial mismatch repair enzyme MutS (51) (Figure 7A). The presence of a G:T mismatch in this substrate kinks the DNA at a 45° angle (Supplementary Figure S10C), allowing it to interact unencumbered with all four basic patches (Figure 7A). Bp^CTR^ does not show any preference for a substrate containing a G:T mismatch in an exonuclease assay (Supplementary Figure S4C and D).

Mutation of positive residues in patch 1 (R552A/K555A) and patch 3 (R467A/R468A) reduces the DNA cleavage activity of Bp^CTR^ on both supercoiled plasmids and linear lambda DNA (Figure S10D and E), thus indirectly implicating these regions as important for binding. R405A (patch 4) and R559A (patch 1) substitutions do not significantly impair the overall cleavage compared to wildtype (Supplementary Figure S10D and E). We do note, however, accumulation of uncut, supercoiled DNA with every mutant (Supplementary Figure S10E), suggesting each region contributes at least partially to orienting DNA in a manner that promotes endonuclease function. A truncation construct deleting the helical bundle helices α1^H^–α5^H^ but retaining the stabilizing α6^H^ helix (Δ505–577) severely impairs both nuclease degradation and nicking (Supplementary Figure S10D and E), further highlighting the importance of this region in DNA binding and catalytic function.

The orientation of the modelled substrate would clash with both the Toprim core α2 and α3 helices in their canonical positions and the Insert 1 segments present in topoisomerases and gyrases (Figure 7B), suggesting that OLD nucleases associate with DNA differently than other Toprim proteins. Importantly, this arrangement places one strand directly into the Bp OLD active site cleft with a phosphate residue situated between metal A and metal B (Figure 7C). K562 is 2.8 Å away from the back side of the scissile phosphate, where it would be primed either to stabilize the charge in the transition state along with metal B and/or protonate the leaving group following cleavage. This favors the proposed catalytic mechanism diagrammed in Figure 7D.

## DISCUSSION

Here we have described the structural and biochemical characterization of the Class 2 OLD proteins from *B. pseudomallei* and *X. campestris* pv. *campestris*. Bp and Xcc OLD catalyze metal-dependent nicking and cleavage of DNA substrates *in vitro*. While the N-terminal region containing the ATPase domain is dispensable for these activities, its presence mediates higher ordered oligomerization of Class 2 OLD proteins (Supplementary Figure S1E and F). We suspect that the ATPase domain may act in a regulatory capacity, controlling how and when the catalytic C-terminal region accesses substrates.

The Bp^CTR^ structure elucidates the catalytic machinery of Class 2 OLD proteins. In addition to the canonical invariant glutamate (E400) and DxD aspartates (D455 and D457), we identify E404, T506 and E508 as side chains that play a role metal binding. These residues are absolutely conserved among Class 2 OLD proteins (Supplementary Figure S7) and together coordinate two bound magnesium ions in a geometry that supports two-metal catalysis (Figures 5A and 7C and D). Single point mutations at these sites are tolerated, whereas triple mutant substitutions removing all metal coordination completely abolish processive degradation of substrates (Figure 5C and D). We speculate that a water may be capable substituting as a ligand when a single metal binding residue is mutated, especially since some of the metal contacts in the Bp^CTR^ crystal are water mediated in the absence of substrate.

We also find K562 in the Bp helical domain is critical for efficient catalytic function (Figure 5). K562 is directed toward the putative scissile phosphate in our Bp^CTR^-DNA bound model (Figure 7C), where it would be poised to stabilize the developing negative charge in the transition state and/or protonate the leaving group. Significant perturbation to one part of the key catalytic machinery (metal A, metal B, or K562) still permits Bp^CTR^ to nick and linearize plasmid DNA; however, processive DNA cleavage is only achieved when the three elements are intact (Figure 5C and D). Truncation of the helical domain (Δ505–577) or simultaneous mutation of both metal sites (2A/2B mutant) impairs both functions (Figure 5C and D, Supplementary Figure S10D and E). Together these results argue that nicking only requires a single metal but full nuclease activity in Class 2 OLD proteins requires proper coordination of two metals and the presence of the conserved lysine. Class 1 OLD proteins are on average ∼50 amino acids shorter and diverge from their Class 2 counterparts in portions of the C-terminal region, which prohibits the unambiguous identification of Class 1 catalytic machinery by sequence alignment alone. Structural and biochemical characterization of the Class 1 OLD homolog from *Thermus scotoducts* indicates that the mechanisms and machinery we describe here for nuclease cleavage is conserved (Schiltz and Chappie, in review).

The spatial organization of acidic residues in the Bp OLD active site directly mirrors that of RNase M5 maturases (Figure 6, Supplementary Figure S9). In addition to conserved catalytic residues previously identified through the biochemical characterization of *Bacillus subtilis* RNase M5 (14), our structural comparison with the available *A. aeolicus* RNase M5 structure suggests that a C-terminal glutamate (E96 in *B. subtilis*; E110 in *A. aeolicus*) will also be critical for 5S RNA maturation. Interestingly, *A. aeolicus* RNase M5 appears to be truncated and circular permutated. Many other homologs including *B. subtilis* contain C-terminal helical extensions (13,14) that could fold into a domain like that observed in Class 2 OLD proteins. DnaG primases also share this conserved arrangement of active site residues (16,17); however, structural constraints imposed by the N-terminal subdomain in the RNA polymerase core prevent the coordination of the Toprim metal B in the same manner. A third metal observed in the *Staphylococcus aureus* DnaG structure (20), which occupies the same position as E508 in Bp OLD, appears to compensate. Importantly, this common active site blueprint is distinct from topoisomerases, gyrases and RecR (Supplementary Figure S9). The overall structural similarity between primases, maturases, and OLD nucleases thus implies a common evolutionary lineage and further segregates the Toprim family into distinct subgroups based on differences in metal coordination, with the distinguishing feature being the presence or absence of additional acidic residues beyond the canonical Toprim glutamate and DxD aspartates.

Our initial biochemistry indicated that Bp^CTR^ and Xcc^CTR^ were more active in Mn^2+^; however, further analysis by ICP-AES analysis revealed that both of the purified constructs preferentially contained bound Ca^2+^ and Mg^2+^ and no Mn^2+^ (Supplementary Table S1). Addition of calcium potentiates Bp^CTR^ activity with magnesium *in vitro*. While calcium typically inhibits most nucleases (2), some enzymes like the *Staphylococcal* nuclease utilize calcium in their active site to cleave DNA (52). Additionally, DNase I is known to be most active in the presence of both magnesium and calcium (53). In the case of DNase I, however, magnesium occupies the active site while calcium binds to other regions of the structure to act as an allosteric enhancer (54). Whether calcium plays a direct role in the active site or modulates activity indirectly, possibly by stabilizing the protein or enhancing DNA binding, remains to be determined. Importantly, the Class 1 OLD homolog from *Thermus scotoductus* exhibits the same general affinity for calcium and magnesium and shows the same stimulatory response (Schiltz and Chappie, in review). This implies that utilization of calcium and magnesium is conserved and functionally relevant among all OLD homologs.

Computational modeling shows that a bent DNA substrate engages all four basic patches on the surface Bp^CTR^ while B form DNA would sterically clash with portions of the Toprim domain (Figure 7A and Supplementary Figure S10B). Mutations in patches 1 and 3 reduce Bp^CTR^ activity on both substrates (Supplementary Figure S10D and E), indirectly supporting a role for these regions in DNA binding. These patches flank the active site cleft and in our model anchor the DNA duplex such that one strand is positioned in the active site with a phosphate situated directly between the two bound magnesium ions (Figure 7). The catalytic K562 sidechain resides in patch 2 and engages the substrate at one end of this cleft. Although mutation of R405 in patch 4 does not significantly alter nuclease activity, we note an observable accumulation of the uncut, supercoiled substrate compared to wildtype (Supplementary Figure S10E). This implies patch 4 partially contributes to orienting DNA in a manner that promotes endonuclease function.

The orientation of DNA suggested by our model differs significantly from how other Toprim proteins engage their substrates. Importantly, the structural constraints of this arrangement explain (i) the lack of an insert 1 in OLD Toprim domains, (ii) the significant shift in the positions of the α2 and α3 Toprim core helices in Bp^CTR^ and Xcc^CTR^ and (iii) the position of the helical domain on the opposite side of the core Toprim fold. In the absence of DNA bound structure, we cannot rule out that substrate binding induces further structural changes in the OLD CTR, including those that would permit the unhindered association with an extended B form DNA duplex. Conformational rearrangements could also be coupled to ATP hydrolysis in the full-length protein.

Our binding model, however, does not preclude Bp OLD from also binding DNA ends. Here the terminal phosphate would become the scissile phosphate. This arrangement is equally compatible with the catalytic machinery and indeed Bp^CTR^ exhibits 5′-3′ exonuclease activity (Supplementary Figure S4). P2 OLD exhibits exonuclease activity *in vitro* (28) and Bp OLD readily degrades linear lambda DNA in the presence of Mn^2+^ or Mg^2+^ and Ca^2+^ as detailed above. Bp and Xcc OLD also can nick and cleave circular plasmids, suggesting a robust endonuclease activity. OLD nucleases thus appear to act as either an endo- or exonuclease depending on the substrate presented (Supplementary Figure S4E). The Mre11 nuclease, which functions in double strand break repair and processing, displays a similar duality: it functions as a 3′-5′ exonuclease on double strand DNA and an endonuclease on single strand DNA at protruding 3′- and 5′-ends and 3′ branches (55–57). Further biochemical characterization will be necessary to determine how these different modes of cleavage contribute to OLD function *in vivo*.

While the role of P2 OLD in bacteriophage lambda interference is well documented (23), little is known about the function of other OLD homologs *in vivo*. Our bioinformatics data indicate that OLD proteins are widely distributed across bacteria, archaea, and viral genomes. The presence of *old* genes in species-specific operons and on mobile elements suggest they confer a functional advantage. We speculate that these proteins may play a novel role in DNA repair and/or replication based the specific association of UvrD/PcrA/Rep helicase with Class 2 OLD proteins. Future genetic experiments will be necessary to validate this hypothesis and define the biological roles of OLD nucleases more explicitly.

## DATA AVAILABILITY

The atomic coordinates and structure factors for the Xcc^CTR^ Hg, Pt, and I derivatives are deposited in the Protein Data Bank with accession numbers 6NJW, 6NJX and 6NJV respectively. The atomic coordinates and structure factors of the Bp^CTR^ structure are deposited in the Protein Databank with the accession number 6NK8.

## Supplementary Material

gkz703_Supplemental_Files
