# Supplementary material for: Structural characterization of Class 2 OLD family nucleases supports a two-metal catalysis mechanism for cleavage
Source: Nucleic Acids Res. 2019 Aug 10;47(17):9448–63. doi: 10.1093/nar/gkz703 (PMC6755086; doi:10.1093/nar/gkz703)
Supplement: gkz703_Supplemental_Files [file gkz703_supplemental_files.zip › bpold_ctr_nar_revision2_supplemental_final.pdf]

Supplementary information for:

Structural characterization of Class 2 OLD family nucleases supports a two-metal catalysis mechanism for cleavage

Carl Schiltz[1], April Lee[1], Edward A. Partlow[1], Christopher J. Hosford[1], and Joshua S. Chappie[1,*]

[1] Department of Molecular Medicine, Cornell University, Ithaca, NY, 14853, USA

* To whom correspondence should be addressed. Tel: +1 (607) 253-3654; Fax: +1 (607) 253-3659; Email: chappie@cornell.edu

Supplementary Tables

Table S1. Inductively coupled plasma atomic emission spectroscopy (ICP-AES) analysis of OLD protein metal binding.

Table S2. X-ray data collection and refinement statistics.

Table S3. Classification of *old* genes and associated operons.

Supplementary Figures

Figure S1. Classification of OLD proteins, purification, and SEC-MALS analysis of Class 2 constructs. Related to Figure 1.

Figure S2. Metal dependence and quantification of Xcc OLD nuclease activity. Related to Figure 1.

Figure S3. Calcium potentiates OLD CTR nuclease activity when coupled with magnesium. Related to Figure 1.

Figure S4. Bp^{CTR} has 5'-3' exonuclease activity. Related to Figures 1 and 7.

Figure S5. Structure of Xcc^{CTR}. Related to Figure 2.

Figure S6. Comparison of OLD CTR structures. Related to Figure 2.

Figure S7. Sequence alignment and conservation of the C-terminal region in Class 2 OLD proteins. Related to Figures 2 and 5.

Figure S8. Structural comparison of Bp^{CTR} with other Toprim domains. Related to Figure 3.

Figure S9. Cartoon depictions of Toprim fold topologies. Related to Figure 3.

Figure S10. Structural constraints of Bp^{CTR} DNA binding. Related to Figure 7.

Table S1. Inductively coupled plasma atomic emission spectroscopy (ICP-AES) analysis of OLD protein metal binding.

Milliequivalents of metal per protein molecule

	**Mg**	**Mn**	**Ca**	**Zn**	**Ni**	**Co**
**Bp^{CTR}**	18.38	-0.38	79.35	13.25	3.21	1.12
**Xcc^{CTR}**	11.39	0.06	100.27	8.29	6.75	-0.01

Table S2. X-ray data collection and refinement statistics.

	Xcc^{CTR} Pt<br>PDB: 6NJW	Xcc^{CTR} Hg<br>PDB: 6NJX	Xcc^{CTR} I<br>PDB: 6NJV	Bp^{CTR}<br>PDB:6NK8
**Data collection**				
Space group	$P4_3$	$P4_3$	$P4_3$	$C\,2\,2\,2_1$
Cell dimensions				
a, *b*, *c* (Å)	65.4, 65.4, 63.8	65.2, 65.2, 63.8	64.4, 64.4, 63.4	83.3, 105.7, 123.8
α, β, γ (°)	90, 90, 90	90, 90, 90	90, 90, 90	90, 90, 90
Resolution (Å)	45.66-1.86 (1.93-1.86)	46.10-1.95 (2.02-1.95)	45.17-2.30 (2.38-2.30)	61.88-2.24 (2.32-2.24)
R_{sym} or R_{merge}	0.07 (1.41)	0.09 (1.56)	0.08 (0.82)	0.07 (1.18)
R_{meas}	0.071	0.092	0.084	0.080
$CC_{1/2}$ (%)	99.7 (52.7)	99.8 (58.7)	99.9 (72.4)	99.9 (54.1)
$I / \sigma I$	19.5 (1.2)	15.8 (1.3)	16.5 (2.2)	18.2 (1.8)
Completeness (%)	99.9 (99.9)	100 (99.9)	99.9 (99.7)	99.4 (97.4)
Redundancy	10.2 (10.4)	10.3 (10.7)	6.7 (6.2)	6.5 (6.4)
**Phasing**				
Initial F.O.M.	0.62	0.64	0.50	
Number of sites	1	3	4	
**Refinement**				
Resolution (Å)	45.66-1.86	46.10-1.95	45.17-2.30	61.88-2.24
No. reflections	22692	19603	11611	26558
R_{work} / R_{free} (%)	21.2/23.8	20.1/24.1	21.5/27.5	21.3/26.0
No. atoms				
Protein	1647	1615	1597	3128
Ligand/ion	4	6	4	6
Water	73	84	41	78
B-factors				
Protein	49.1	51.8	56.5	30.6
Ligand/ion	119.4	50.5	65.6	25.9
Water	49.5	51.9	54.3	21.4
R.m.s deviations				
Bond lengths (Å)	0.008	0.01	0.009	0.008
Bond angles (°)	1.20	1.32	1.35	1.24
**Ramachandran statistics**				
Favored (%)	97	97.0	93.6	97.2
Allowed (%)	3	3.0	6.4	2.5
Outliers (%)	0	0	0	0.3

*Values in parentheses are for highest-resolution shell. Each dataset was derived from a single crystal.

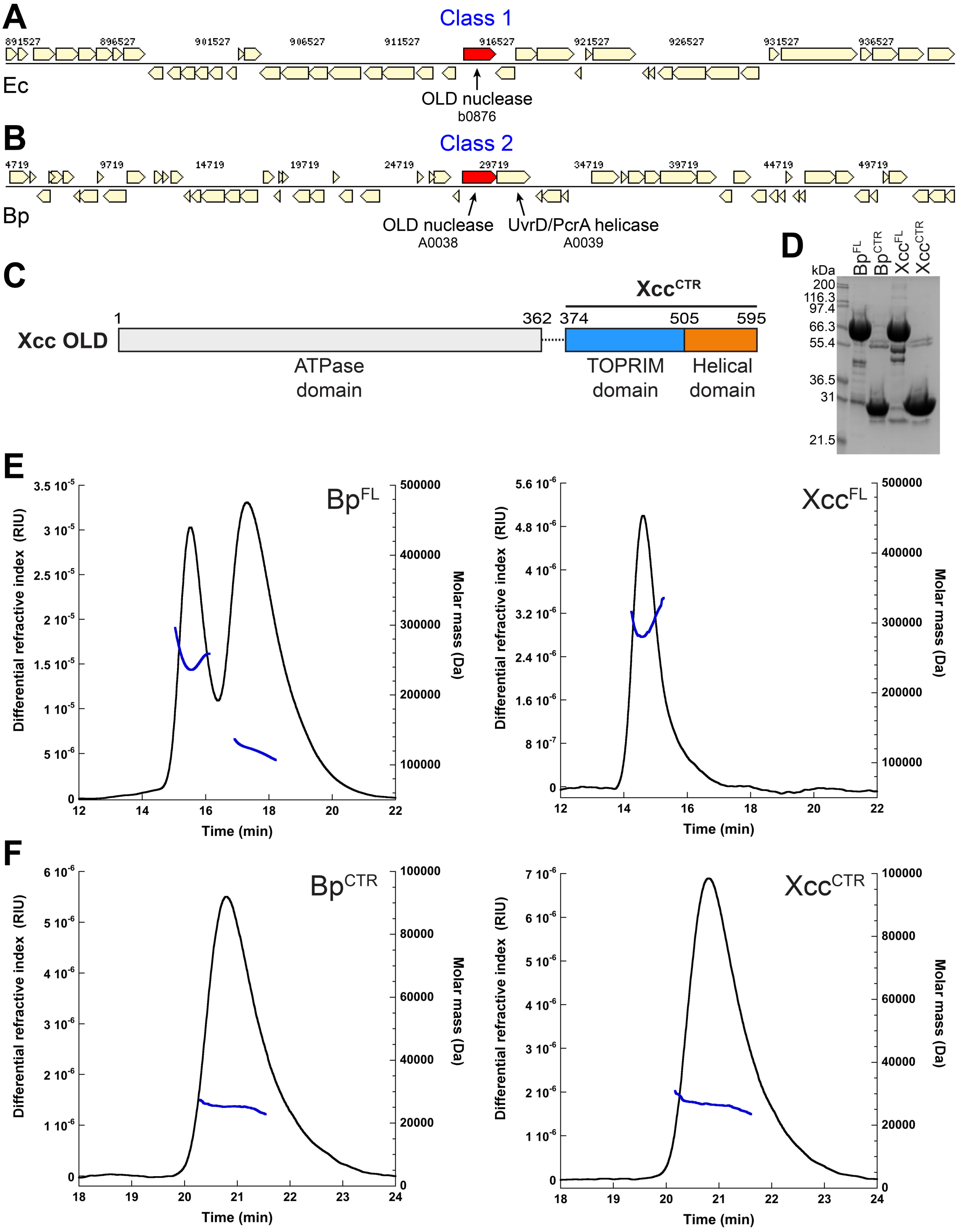

Figure S1. Classification of OLD proteins, purification and SEC-MALS analysis of Class 2 constructs. A-B. Gene neighborhoods of Class 1 OLD homolog from *Escherichia coli* (A) and Class 2 OLD homolog from *Burkholderia Burkholderia pseudomallei* (B). Gene arrangements and annotations derived from DOE IMG/M database and analysis tools (40). **C.** Domain architecture of the Xcc OLD protein with construct boundaries marked. **D.** SDS-PAGE gel showing purified Bp^{FL}, Bp^{CTR},

Xcc^{FL}, and Xcc^{CTR}. **E-F.** SEC-MALS analysis of Bp and Xcc FL (E) and CTR (F) constructs. Black line denotes differential refractive index and blue line denotes measured mass across each peak.

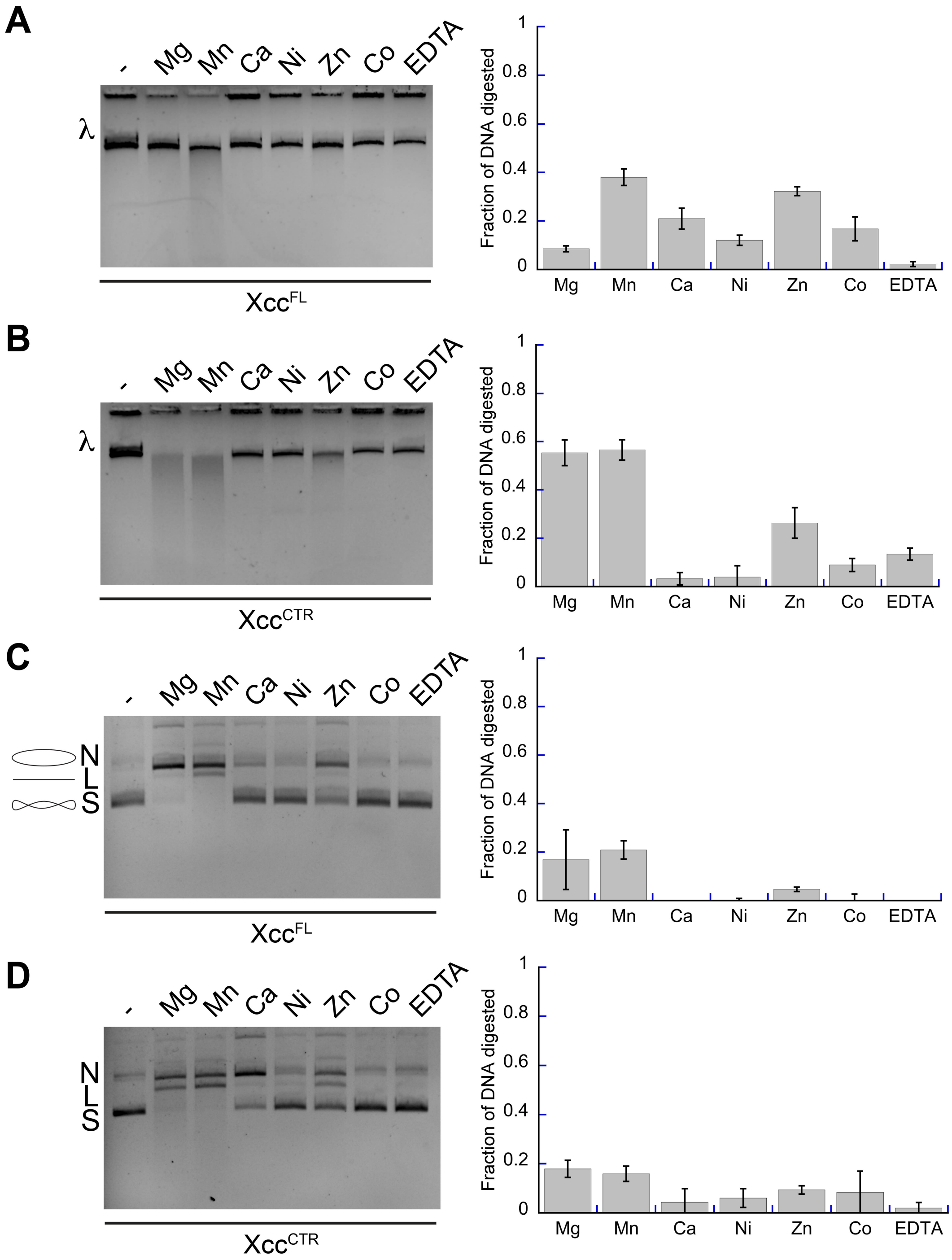

Figure S2. Metal dependence and quantification of Xcc OLD nuclease activity. A-B. Representative agarose gel and quantification of metal-dependent nuclease activity of Xcc^{FL} (A) and Xcc^{CTR} (B) on linear lambda phage DNA (λ). **C-D.** Representative agarose gel and quantification of metal-dependent nuclease activity of Xcc^{FL} (C) and Xcc^{CTR} (D) on supercoiled pUC19 plasmid DNA. Supercoiled (S), nicked (N), and linear (L) pUC19 DNA is denoted next to the gels. Lanes labeled with dashed lines indicate samples with no metal added. In all instances, DNA degradation was quantified

using BioRad Image Lab software as described in the Materials and Methods. Bar graphs represent the average of three independent experiments with error bars representing the standard error of the mean.

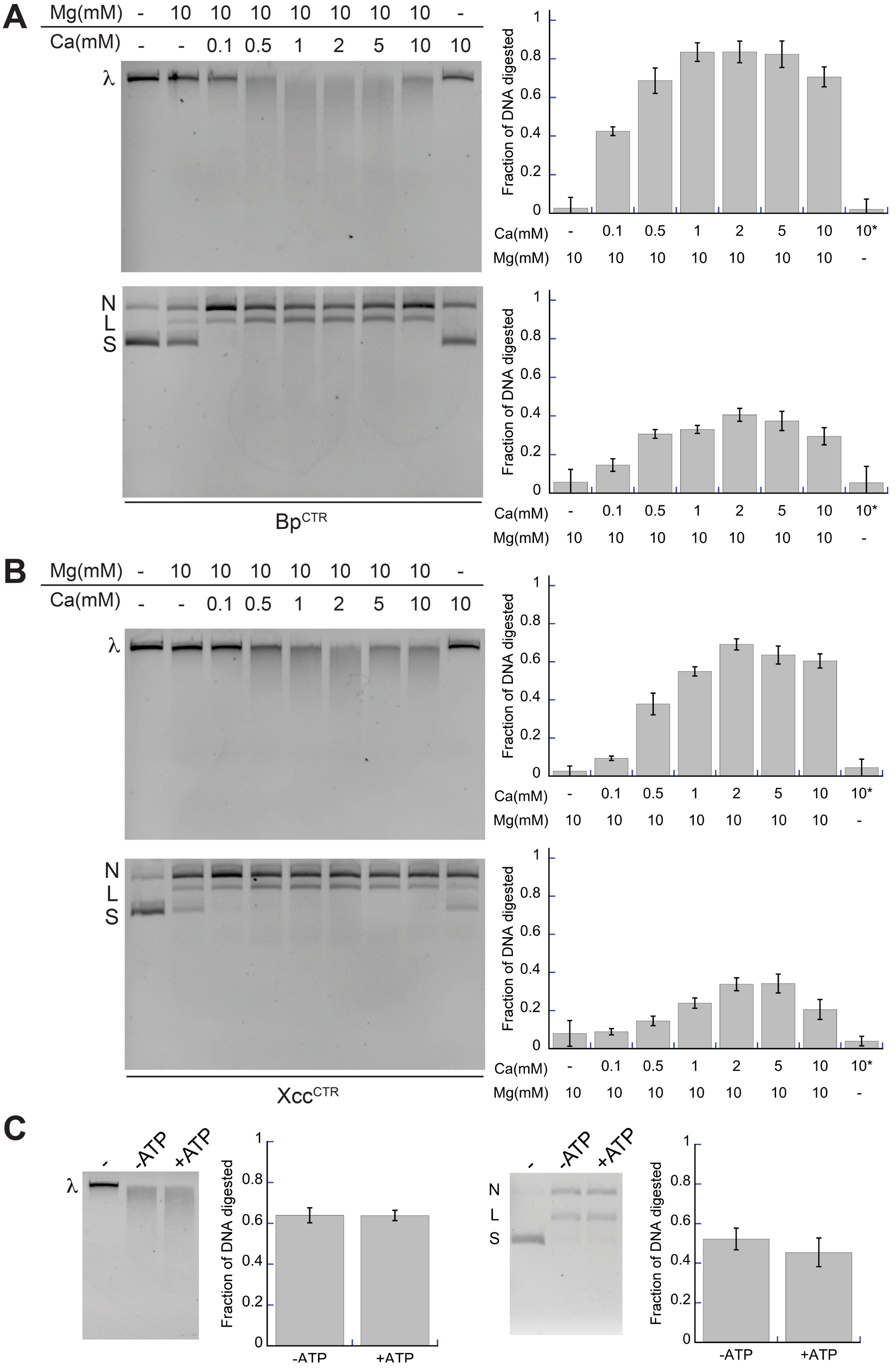

Figure S3. Calcium potentiates OLD CTR nuclease activity when coupled with magnesium. A. Representative agarose gel and quantification of Bp^{CTR} nuclease activity on linear lambda DNA (λ) and supercoiled pUC19 DNA (S) with a constant concentration of magnesium (10mM) and increasing

amounts of calcium. **B.** Representative gel and quantification of Xcc^{CTR} nuclease activity on linear and supercoiled DNA with magnesium and increasing amount of calcium. **C.** Measurement of Bp^{FL} nuclease activity on linear and supercoiled DNA in the present or absence of ATP. Lanes labeled with dashes indicate substrate alone controls without protein added. In all instances, activity was quantified using BioRad Image Lab software as described in the Materials and Methods. Bar graphs represent the average of three independent experiments with error bars representing the standard error of the mean.

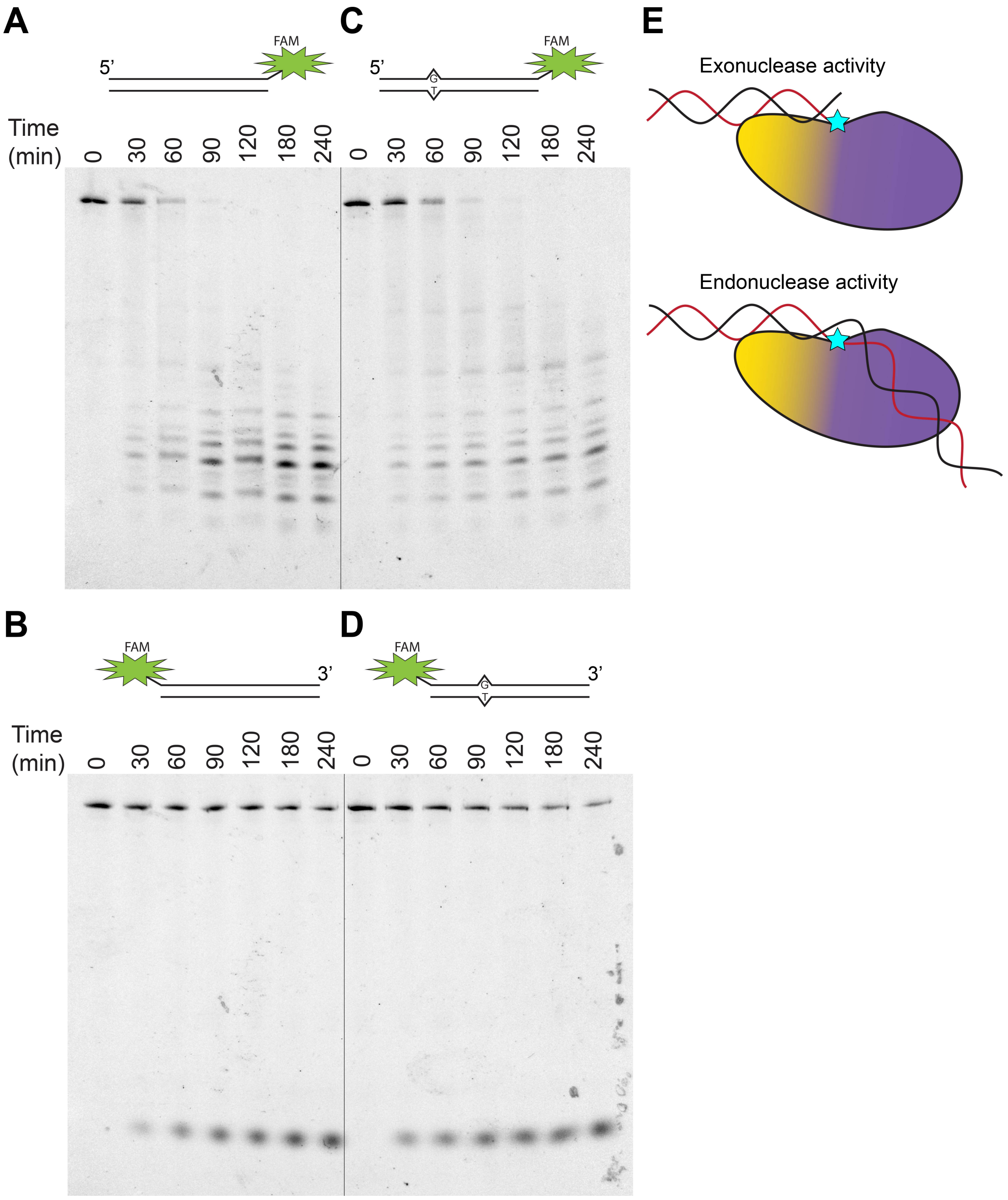

Figure S4. Bp^{CTR} has 5'-3' exonuclease activity. A-B. Time course of Bp^{CTR} digestion of a 40 bp double-stranded DNA substrate containing either a 3' (A) or 5' (B) 6-carboxyfluorescein (6-FAM) label. **C-D**. Time course of Bp^{CTR} digestion of a G-T mismatched 40 bp double stranded DNA substrate containing either a 3' (C) or 5' (D) 6-FAM label. Mismatched substrates in A and B are intended to mimic the bent DNA substrate modeled in Figure 7A. **C**. Cartoon modeling how Bp^{CTR} could support exonuclease activity on a blunt substrate as well as nicking and endonuclease activity on supercoiled

dsDNA. DNA strands are colored red and black. Purple and yellow indicate relative positions of Toprim and helical domains. Cyan star denotes position of scissile phosphate.

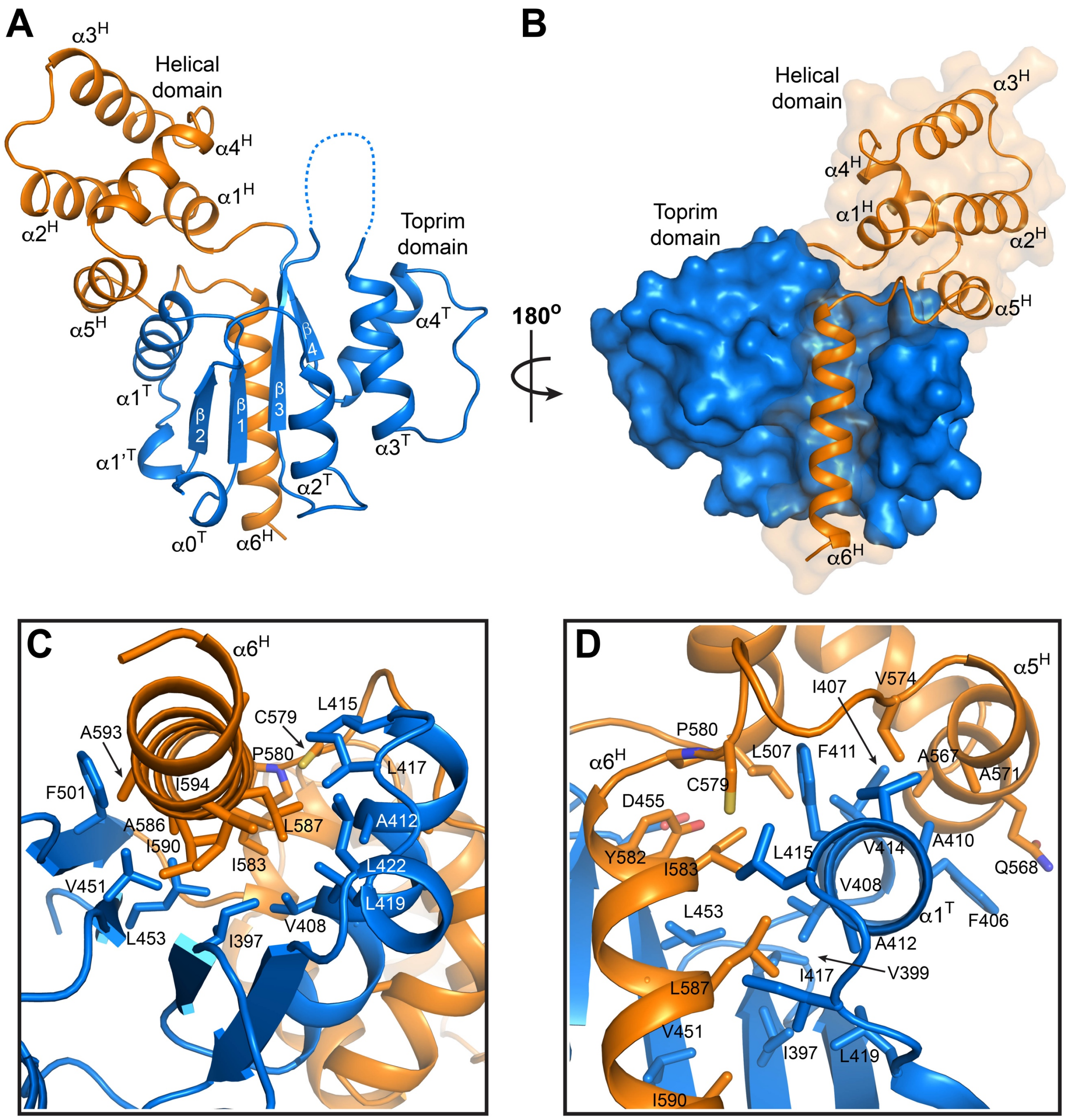

Figure S5. Structure of Xcc^{CTR}. **A**. Structure of Xcc^{CTR}. Toprim and helical domains are blue and orange respectively. **B**. Surface representation of domain interactions. **C-D**. Structural interactions between Toprim and helical domains. Side chains involved in stabilizing hydrophobic interactions are labelled.

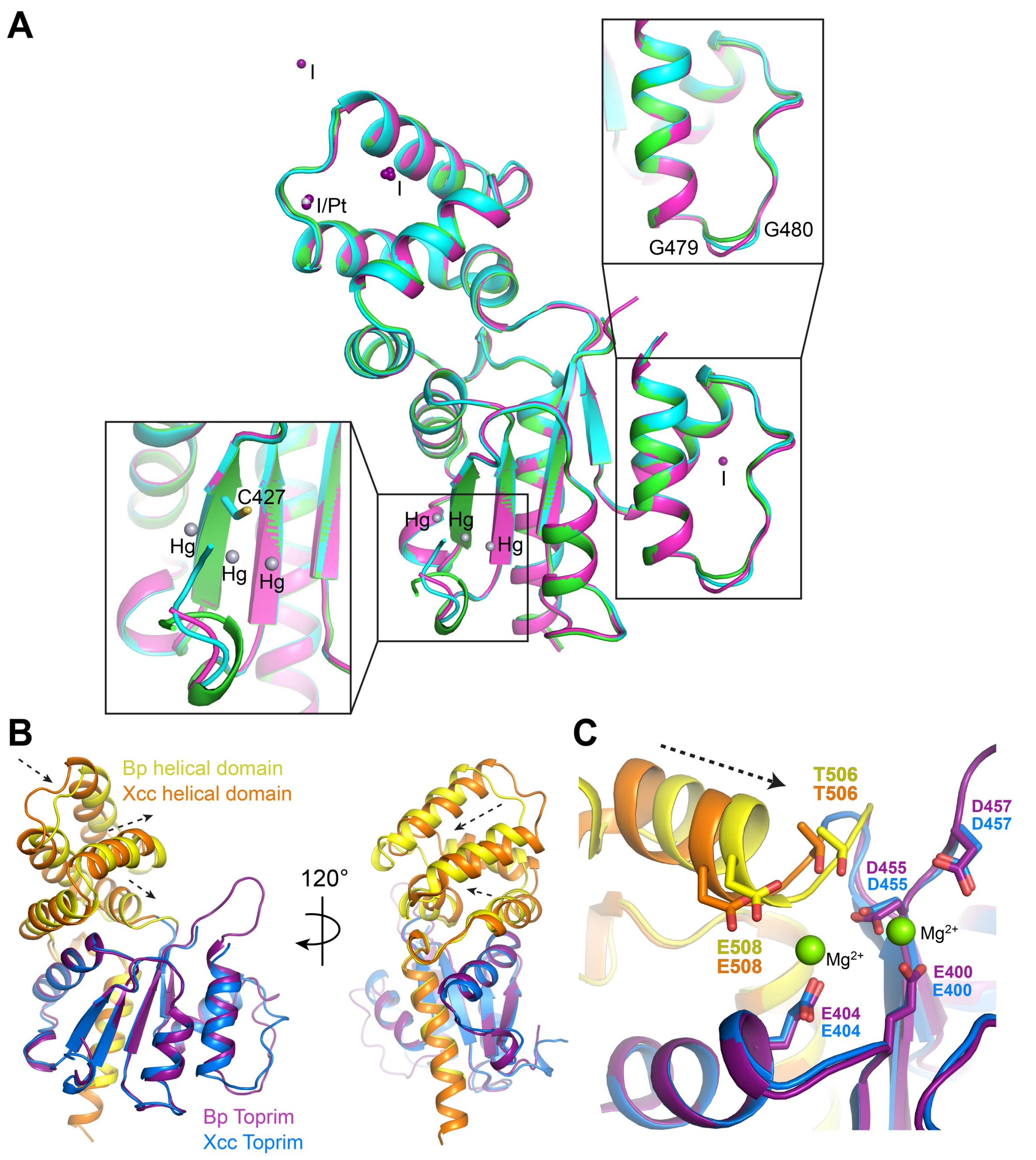

Figure S6. Comparison of OLD CTR structures. A. Structural superposition of Xcc^{CTR} derivative models. Pt, Hg, and I derivatives solved by SAD phasing are colored green, cyan, and magenta respectively. Zoomed inserts show minor structural differences between models. **B-C.** Structural superposition of Xcc^{CTR} (orange and blue) and Bp^{CTR} (yellow and purple. Movement of the Bp OLD helical domain relative to the Toprim domain (B, dashed arrows) shifts the glutamate helix toward the active site (C, dashed arrow), thereby facilitating the binding of two magnesium ions (C, green spheres).

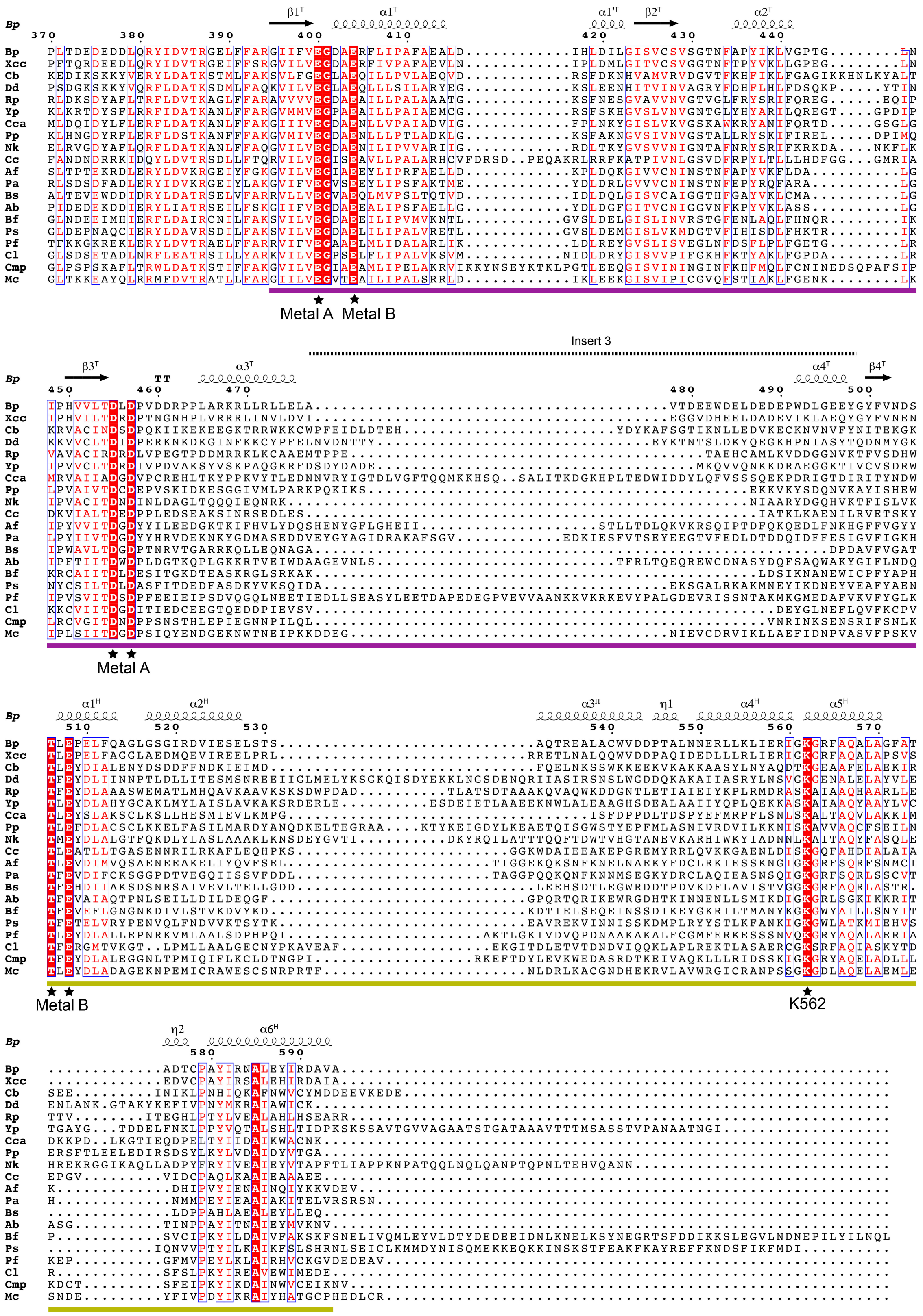

Figure S7. Sequence alignment and conservation of the C-terminal region in Class 2 OLD proteins. Secondary structure of Bp^{CTR} indicated above sequences. Purple and yellow bars below the sequences denote the Toprim and helical domain boundaries respectively. Insertion into the core

Toprim fold present in Bp and Xcc OLD (Insert 3) is labeled (see Figure 3 and Figures S7 and S8). Stars denote positions of metal A and metal B coordinating residues and K562. Sequence shading indicates conservation: white text on red background, 100% conserved; boxed red text on white background, 70% conserved. Abbreviations are as follows: Bp, *Burkholderia pseudomallei*; Xcc, *Xanthamonas campestris* pv. *campestris* B100; Cb, *Clostridium botulinum* A Hall; Dd, *Desulfitobacterium dichloroeliminans*; Rp, *Rhodococcus pyridinivorans*; Yp,*Yersinia pseudotuberculosis* YPIII; Cca, *Candidatus Cloacimonas acidaminovorans*; Pp, *Paenibacillus peoriae*; Nk, *Niastella koreensis*; Cc, *Corynebacterium callunae*; Af, *Anoxybacillus flavithermus*; Pa, *Peptoclostridium acidaminophilum*; Bs, *Blastococcus saxobsidens*; Ab, *Acinetobacter baumannii* AB0057; Bf, *Bacteroides fragilis* NCTC9343; Ps, *Psychromonas* sp. CNPT3; Pf, *Pseudomonas fluorescens* LBUM223; Cl, *Chlorobaculum limnaeum*; Cmp, *Candidatus Methanosphaerula palustris*; Mc, *Methanosaeta concilii*.

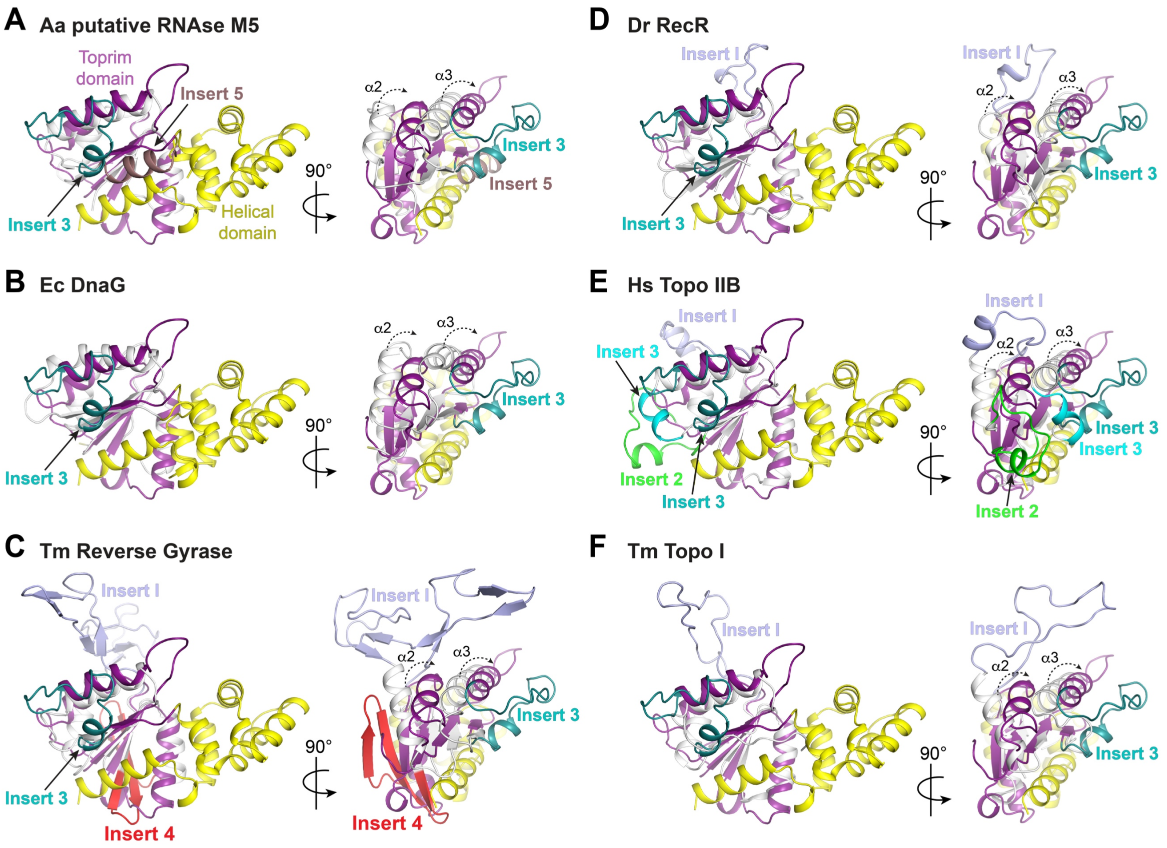

Figure S8. Structural comparison of Bp^{CTR} with other Toprim domains. A-F. Superposition of BpCTR with *Aquifex aeolicus* putative ribonuclease M5 (PDB: 1T6T) (A), *Escherichia coli* DnaG primase (PDB: 3B39) (B), *Thermotoga maritima* reverse gyrase (PDB: 4DDU) (C), *Deinococcus radiodurans* RecR (PDB: 1VVD) (D), *Homo sapiens* Topoisomerase IIB (PDB: 3QX3) (E), and *Thermotoga maritima* Topoisomerase I (PDB: 2GAJ) (F). Bp Toprim and helical domains are colored purple and yellow respectively while the corresponding Toprim cores of all other proteins are colored gray. Structural elements inserted into the minimal core fold are individually labeled and colored (Inserts 1-5). Side (left) and end (right, rotated 90°) views are shown for each superposition. Dashed arrows indicate α2 and α3 helical shifts in Bp OLD Toprim core relative to the Toprim central β-sheet.

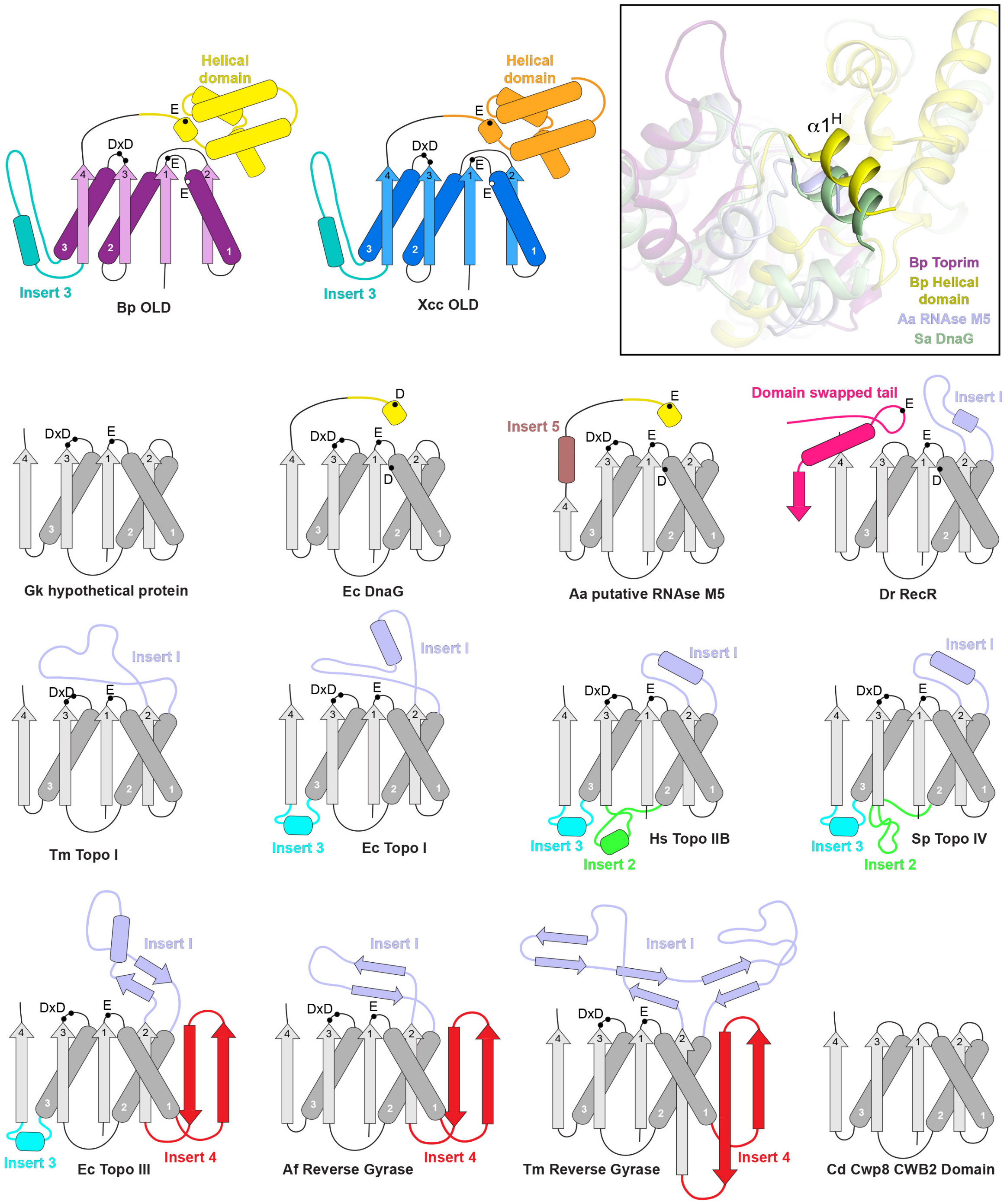

Figure S9. Cartoon depictions of Toprim fold topologies. Bp and Xcc Toprim cores are shown in purple and blue respectively while the corresponding cores of all other proteins are colored gray. β-sheets are depicted as thick arrows while helices are shown as rounded cylinders, Numbering indicates order and sequence trajectory. Structural elements inserted into the minimal core fold are individually labeled and colored (Inserts 1-5). The Bp and Xcc helical domains are shown as distinct inserts, colored yellow and orange respectively. C-terminal helix present in primases and maturases

is shown in yellow. Conserved active site residues are labeled in each model. Abbreviations as follows: Gk,*Geobacillus kaustophilus*; Ec, *Escherichia coli*; Aa, *Aquifex aeolicus*; Dr, *Deinococcus radiodurans;* Tm, *Thermotoga maritima*; Hs, *Homo sapiens*; Sp, *Streptococcus pneumoniae*; Af, *Archaeoglobus fulgidus*; Cd, *Clostridium difficile*. Boxed inset at top right highlights structural conservation of C-terminal helix in RNase M5 maturases and DnaG primases with the aH1 helix in Bp OLD. Superposition includes the Aa RNase M5 (PDB: 1T6T) and *Staphylococcus aureus* (Sa) DnaG (PDB: 4EE1).

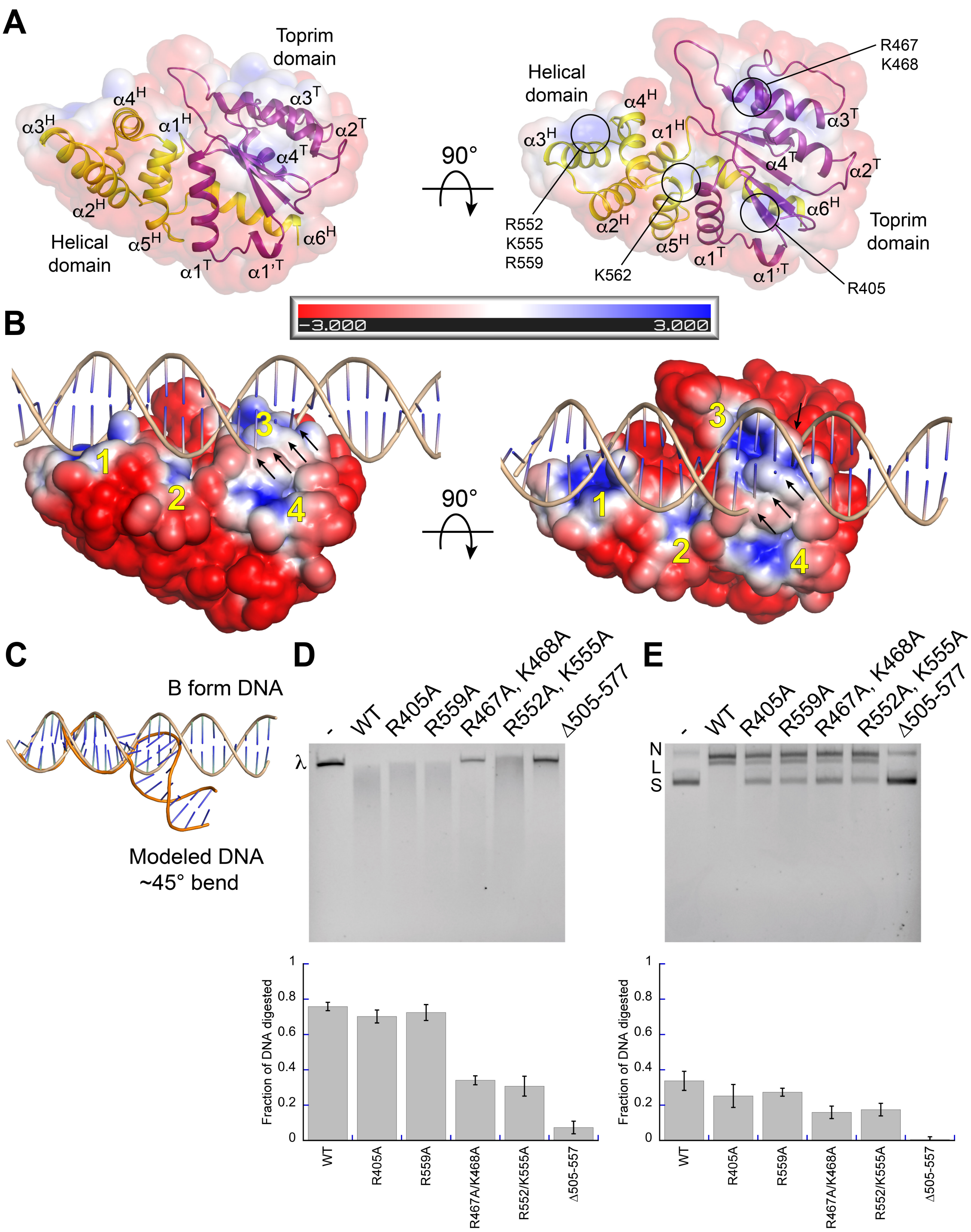

Figure S10. Structural constraints of Bp^{CTR} DNA binding. A. Domain contribution to electrostatic surface and DNA binding. Electrostatic surface of Bp^{CTR} rendered semi-transparent to reveal relative positions of Toprim (purple) and helical (yellow) domains. Electrostatic potential calculated with APBS (37). Scale indicates coloring of the potential from -3 K_bT/e_c to +3 K_bT/e_c. Black circles denote relative location of the four basic patches numbered in B. Residues contributing these basic patches are labeled. **B.** Electrostatic surface of Bp^{CTR} shown with modeled B-form DNA and colored as in A. The

four basic patches around the active site cleft are numbered in yellow. Black arrows indicate steric clashes. **C.** Modeled DNA substrate in Figure 7A is significantly bent relative to B-form DNA. **D-E.** Nuclease assay of Bp^{CTR} with mutations in proposed DNA binding patches with linear (D) and supercoiled substrates (E). Mutations R559A and R552A/K555A are located in Patch 1, mutations R467A/R468A are in Patch 3, and R405A is in Patch 4. The catalytic K562 sidechain constitutes Patch 2 (see Figures 5 and 7C-D). The Bp^{CTR} mutant Δ505-577 denotes deletion of the helical bundle ($\alpha 1^H$-$\alpha 5^H$). DNA degradation in D and E was quantified using BioRad Image Lab software as described in the Materials and Methods. Bar graphs represent the average of three independent experiments with error bars representing the standard error of the mean.
